# CRISPR-based knockout and base editing confirm the role of MYRF in heart development and congenital heart disease

**DOI:** 10.1242/dmm.049811

**Published:** 2023-08-16

**Authors:** Lino Doering, Alex Cornean, Thomas Thumberger, Joergen Benjaminsen, Beate Wittbrodt, Tanja Kellner, Omar T. Hammouda, Matthias Gorenflo, Joachim Wittbrodt, Jakob Gierten

**Affiliations:** ^1^ Centre for Organismal Studies, Heidelberg University, 69120 Heidelberg, Germany; ^2^ Department of Pediatric Cardiology, University Hospital Heidelberg, 69120 Heidelberg, Germany; ^3^ Heidelberg Biosciences International Graduate School, Heidelberg University, 69120 Heidelberg, Germany; ^4^ DZHK (German Centre for Cardiovascular Research), partner site Heidelberg/Mannheim, 69120 Heidelberg, Germany

**Keywords:** CHD, CRISPR-Cas9, Base editing, MYRF, Medaka

## Abstract

High-throughput DNA sequencing studies increasingly associate DNA variants with congenital heart disease (CHD). However, functional modeling is a crucial prerequisite for translating genomic data into clinical care. We used CRISPR-Cas9-mediated targeting of 12 candidate genes in the vertebrate model medaka (*Oryzias latipes*), five of which displayed a novel cardiovascular phenotype spectrum in F0 (crispants): *mapre2*, *smg7*, *cdc42bpab*, *ankrd11* and *myrf*, encoding a transcription factor recently linked to cardiac-urogenital syndrome. Our *myrf* mutant line showed particularly prominent embryonic cardiac defects recapitulating phenotypes of pediatric patients, including hypoplastic ventricle. Mimicking human mutations, we edited three sites to generate specific *myrf* single-nucleotide variants via cytosine and adenine base editors. The Glu749Lys missense mutation in the conserved intramolecular chaperon autocleavage domain fully recapitulated the characteristic *myrf* mutant phenotype with high penetrance, underlining the crucial function of this protein domain. The efficiency and scalability of base editing to model specific point mutations accelerate gene validation studies and the generation of human-relevant disease models.

## Teaser

**Summary:** *In vivo* modeling revealed that cardiac development crucially depends on the transcription factor MYRF. Changes in single nucleotides in conserved protein domains of MYRF cause severe heart defects.

## INTRODUCTION

Congenital heart disease (CHD) is the most common congenital malformation, affecting ∼1% of live births. CHD occurs as an isolated heart defect or with various extracardiac phenotypes, causes high mortality in patients and entails a significant health burden for the entire family. Large-scale sequencing studies have significantly enhanced the discovery rate of the diverse genetic contributors by associating DNA variants with different CHD phenotypes (Gordon et al., 2022; Homsy et al., 2015; Jin et al., 2017; Krane et al., 2021; Reuter et al., 2020; Richter et al., 2020a; Sifrim et al., 2016; Zaidi et al., 2013). However, insufficient experimental validation limits our understanding of the functional role of these variants. Therefore, scalable experimental modeling approaches and precise gene-editing techniques are needed to reliably test and validate the ever-increasing number of new candidate genes and address specific mutations. Converting the association of newfound CHD genetic factors into causality is essential to uncover new disease mechanisms and translate new findings into clinics, improve genetic testing and provide entry points for novel CHD therapies.

Resolving the genotype–phenotype map of CHD is particularly challenged by a marked clinical heterogeneity of cardiac defects arising from the multifactorial origin and polygenic interactions, variable expressivity and incomplete penetrance of DNA variants (Reuter et al., 2020). Given that significant enrichment or independent confirmation of gene variants is currently limited in human studies and would require substantially increased study cohort sizes, the experimental confirmation in model systems is required to define causality and enable translation into clinical care.

In light of this gap, we functionally studied novel CHD variants in the small medaka fish (*Oryzias latipes*), an established biomedical vertebrate model ideally suited for genetic interference and developmental assays at high throughput and low cost (Cornean et al., 2022; Gierten et al., 2020; Hammouda et al., 2021; Meyer et al., 2020; Stemmer et al., 2015). Rapid embryonic development allows fast phenotypic readout, and visualization of the entire cardiovascular system is straightforward in optically transparent teleost embryos. Fish embryos are not immediately dependent on a functional cardiovascular system through passive oxygen supply (Schwerte et al., 2006), offering a unique condition for studying severe heart defects. The high efficiency of CRISPR-Cas9-mediated gene targeting can enable phenotype assessment already in the injected generation (F0) (Hammouda et al., 2021; Hoshijima et al., 2019; Thumberger et al., 2022; Wu et al., 2018). Base editing (Koblan et al., 2018; Richter et al., 2020b; Thuronyi et al., 2019) has recently been employed in zebrafish (*Danio rerio*) and medaka to directly model the phenotypic effect of single-nucleotide variants (SNVs) associated with human disease (Cornean et al., 2022; Rosello et al., 2021). Despite anatomical differences between the four-chambered mammalian and the two-chambered fish heart, high genetic conservation (Howe et al., 2013; Kasahara et al., 2007) and similarities of vertebrate cardiogenesis from fish to mammals (Bakkers, 2011) make principles of developmental phenotypes in fish highly informative and translatable to human heart disease.

We present a combined *in silico* prioritization and *in vivo* modeling approach for functional dissection of CHD candidate variants from gene to single-variant level: efficient CRISPR-based interrogations of cardiac phenotypes in medaka that permit highlighting critical genes in F0 embryos (crispants), and base editing to further dissect specific genotype–phenotype correlations at the level of missense mutations. Of 200 reported genes derived from multiple CHD sequencing studies, our *in silico* prioritization and filtering yielded 12 genes (*RABGAP1L*, *MAPRE2*, *KLHL26*, *CDC42BPA*, *DLX6*, *DYRK1A*, *SMG7*, *MYRF*, *IQGAP1*, *EVC2*, *MLF1* and *ANKRD11*), which we subjected to CRISPR-Cas9-mediated targeted gene inactivation in medaka. Screening crispants highlighted several genes with significant phenotypic effects in the heart and extracardiac morphological aberrations to variable degrees. We further focused on a pronounced cardiac phenotype found in medaka crispants of *myrf*, encoding a transcription factor that has recently been implicated in human cardiac-urogenital syndrome (CUGS; Rossetti et al., 2019). We established a stable *myrf* mutant line that recapitulates the principal features of the crispant phenotype. The hypoplastic embryonic heart and aberrant looping phenotype resemble critical phenotypic heart malformations found in human *MYRF*-associated disease. Our results highlight the crucial role Myrf plays in cardiac development and functional integrity, and the established mutant line enables detailing disease mechanisms and screening potential targets for therapies. Using cytosine and adenine base editing, we engineered specific *myrf* mutations at conserved residues comparable to human missense mutations, functionally establishing the clinical relevance of patient-associated *MYRF* point mutations *in vivo*.

## RESULTS

### F0 CRISPR-Cas9-based targeted gene inactivation screening reveals developmental phenotypes of human CHD candidate genes

**Fig. 1.**
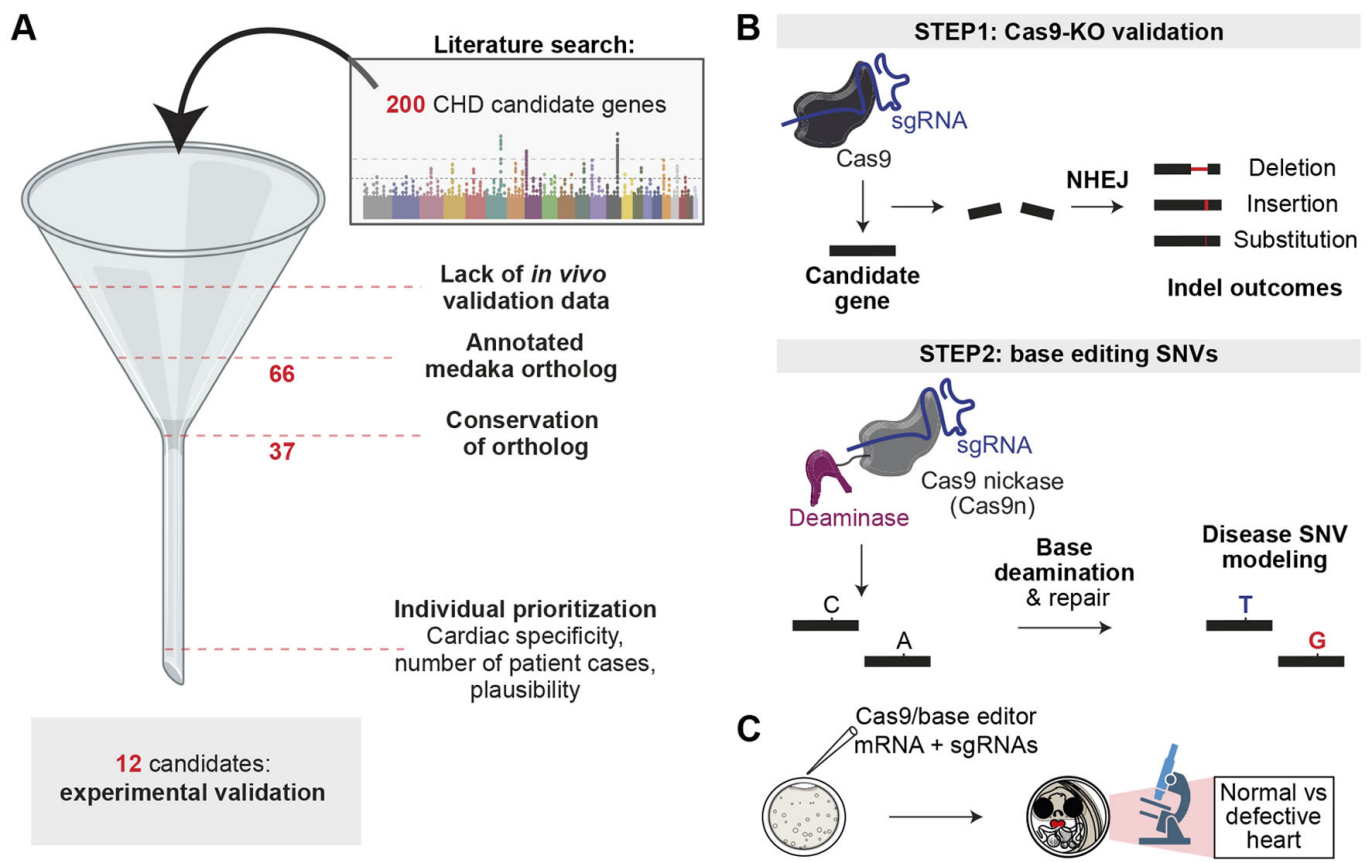
**Selection of human CHD-associated genes and *in vivo* validation procedure in medaka.** (A) Filtering process of candidate genes associated with CHD. (B) Sequential gene assessment with CRISPR-Cas9-mediated targeted gene inactivation for initial validation of candidate genes (step 1) and modeling of SNVs using base editing (step 2). (C) Experimental layout for CRISPR-Cas9-mediated targeted gene inactivation. CHD, congenital heart disease; KO, knockout; NHEJ, non-homologous end joining; sgRNA, single-guide RNA; SNV, single-nucleotide variant. Parts of A and C were created with BioRender.com.

**Fig. 2.**
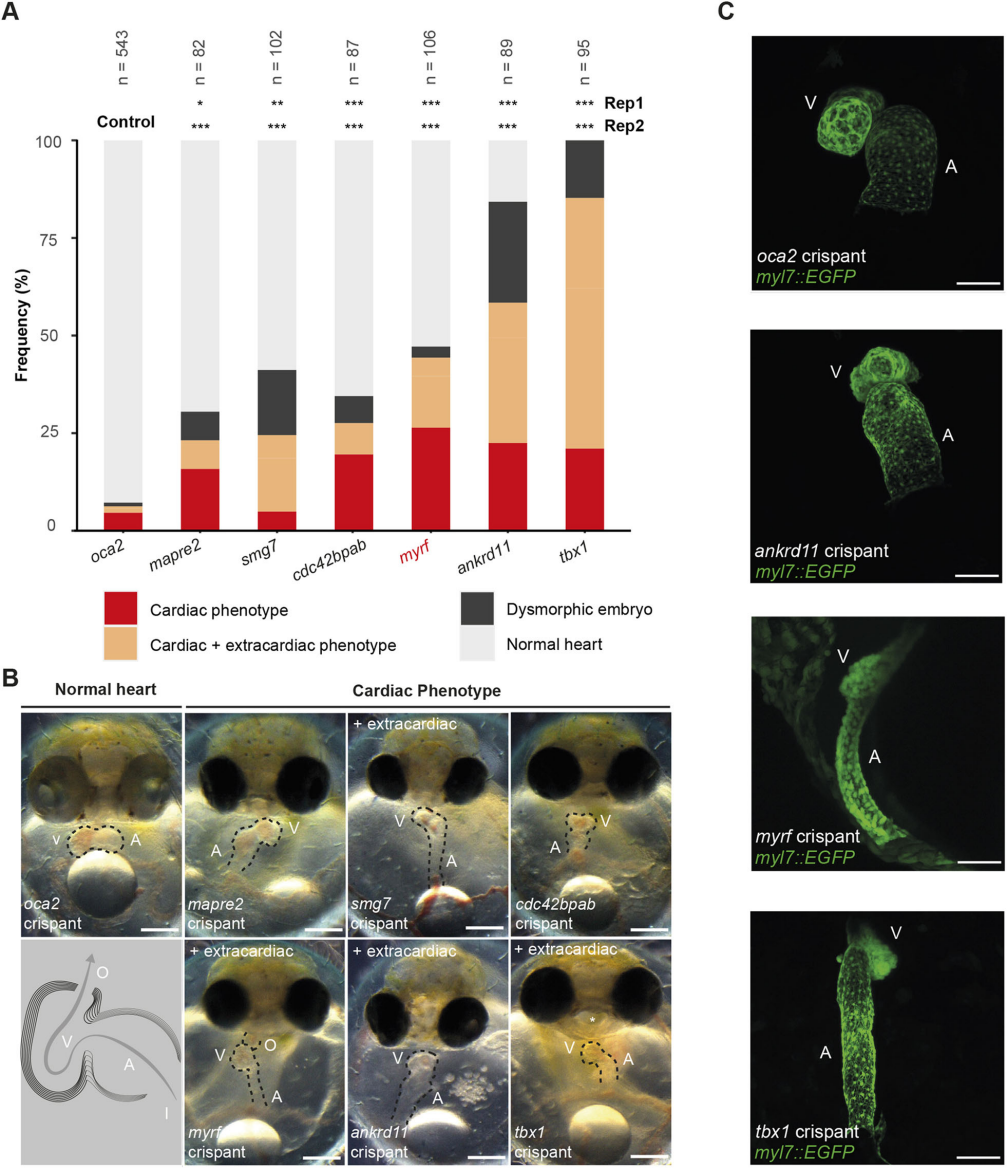
**Embryonic phenotype profiles of preselected CHD candidate genes highlight *myrf* as a critical heart development gene in medaka crispants.** (A) Summary of observed phenotypes at 6 days post fertilization (dpf) per targeted gene (two pooled replicates). *oca2* served as negative control, setting the baseline for injection-procedure-induced phenotypes (pooled replicates, see Materials and Methods). Five of 12 CHD genes displayed cardiac phenotypes in crispants. *tbx1* with known heart and extracardiac functions served as a positive control to capture and discriminate cardiac and extracardiac phenotypes in F0 CRISPR-Cas9-based gene targeting. Statistics given per replicate versus *oca2* control as a Fisher’s exact test (*P<0.05, **P<0.005, ***P<0.0005); phenotyping criteria are defined and exemplified in more detail in Figs S1 and S2. (B) Representative medaka crispants highlighting morphological criteria used for phenotyping. Dashed lines indicate the heart circumference; asterisk indicates craniofacial dysmorphia in *tbx1* embryos. (C) Confocal imaging of crispants in a *myl7::eGFP myl7::H2A-mCherry* background. Looping defects and abnormal atrial or ventricular morphology in 9/10 *ankrd11*, 8/9 *tbx1*, 0/5 *oca2* crispants; *myrf* crispants showed heart looping defects in 4/4 specimens, and 2/4 embryos showed small heart chambers versus 0/4 with structural changes in the *oca2* control group. *oca2*, *tbx1*, *ankrd11*, ventral view; *myrf*, lateral view at 6 dpf; bright-field images were cropped from the original frame and equally adjusted for contrast in Fiji/ImageJ using the brightness and contrast function. Confocal images of *ankrd11*, *oca2*, *tbx1* crispants inverted on the vertical axis; confocal image of *myrf* crispant rotated by 90°. Scale bars: 100 µm for confocal images, 200 µm for bright-field images. A, atrium; crispant, embryo injected with a CRISPR-Cas9 construct at one-cell stage; I, inflow tract; O, outflow tract; Rep, replicate; V, ventricle.

Large-scale sequencing studies of human CHD are an invaluable resource for experimental approaches to uncover new gene functions and identify disease-relevant DNA variants. We retrieved a collection of 200 reported candidate genes from 30 recent CHD sequencing studies and prioritized these candidates *in silico* (Fig. 1A; Tables S1 and S2) to generate a shortlist of genes with expected novelty and a high probability of cardiac relevance. Of these 200 genes, 66 lacked previous *in vivo* experimentation that would highlight a role in cardiac development. Only candidate genes with one conserved ortholog in medaka passed further selection. The remaining candidates were individually assessed regarding the strength of the association with congenital human heart phenotypes, cardiac specificity of reported phenotypes and prior experimental links to heart function. The filtering resulted in 12 genes (*RABGAP1L*, *MAPRE2*, *KLHL26*, *CDC42BPA*, *DLX6*, *DYRK1A*, *SMG7*, *MYRF*, *IQGAP1*, *EVC2*, *MLF1* and *ANKRD11*), which we subjected to Cas9-mediated targeted gene inactivation. Single-guide RNAs (sgRNAs) targeting the medaka ortholog for each candidate gene were microinjected together with Cas9 mRNA into one-cell-stage medaka embryos (Fig. 1B). In these crispants, morphological phenotypes were assessed by bright-field microscopy at 6 days post fertilization (dpf), when cardiovascular development is mainly completed (Fig. 1C). Cardiac phenotypes were identified based on standardized phenotypic features: looping defect, dextrocardia, small ventricle, streaky heart, atrioventricular block, no blood flow or retrograde blood flow in the heart (Fig. 2; Fig. S1). Further, based on extracardiac manifestations, embryos were classified into three groups (isolated cardiac phenotype, cardiac and extracardiac phenotype, and overall dysmorphic embryos; Fig. S1). Genes with highly penetrant cardiac phenotypes upon targeting in F0 were assessed by confocal microscopy using a medaka dual-fluorescent heart reporter line *myl7::EGFP, myl7::H2A-mCherry* (Hammouda et al., 2021).

To account for potential microinjection-induced cardiac phenotypic abnormalities, we compared candidate crispant results to control crispants of the *oculocutaneous albinism type 2* (*oca2*) gene, for which no role in heart formation or performance has been reported (Hammouda et al., 2021; Thumberger et al., 2022). In contrast to the high-penetrant eye pigmentation loss in the *oca2* crispants, cardiac aberrations occurred at a frequency of 6%, setting a low baseline for an injection-induced phenotype rate. Moreover, we included the T-box transcription factor gene *tbx1* as a positive control, owing to its previous syndromic cardiovascular disease associations (Du et al., 2020; Page et al., 2019; Yagi et al., 2003). Crispants of *tbx1* displayed cardiac phenotypes, frequently disturbed cardiac looping, and small ventricles, as well as craniofacial dysmorphia (Fig. 2), reflecting *TBX1* mutation-associated human phenotypes and ventricular morphology and looping defects in zebrafish *tbx1* mutants (Choudhry and Trede, 2013). The F0 CRISPR-Cas9-mediated targeted gene inactivation pinpointed significant phenotypic effects, including cardiac and extracardiac morphological phenotypes. We observed substantial aberrations and reproducible effects (two replicate injections) in five of 12 investigated CHD genes (*mapre2*, *smg7*, *cdc42bpab*, *myrf* and *ankrd11*) (Fig. 2; Fig. S1 and Fig. S2A,B). All test genes with a significant increase in cardiac phenotypes (isolated cardiac phenotypes, and cardiac and extracardiac phenotypes combined; Figs S1 and S2) compared to control showed affected heart looping at different frequencies as well as variable structural heart defects and extracardiac dysmorphia, the latter of which was most pronounced for *ankrd11* and *tbx1*.

The targeting of *ankrd11*, a chromatin modifier involved in histone acetylation, with implications for neurogenesis and autism spectrum disorder (Gallagher et al., 2015) and craniofacial development (Roth et al., 2021), induced cardiac defects in 58% of surviving crispants. Defective heart looping, apparently a sensitive phenotypic readout for most of the targeted candidate genes, was also most frequent in *ankrd11* crispants. Confocal microscopy also highlighted ventricular morphology defects (Fig. 2C). In line with the expected extracardiac epigenetic function of *ankrd11*, crispants displayed a high rate of extracardiac manifestations, with highly increased levels of global dysmorphia, in contrast to the *oca2* control. Although the overall composition of cardiac and extracardiac phenotypes varied widely across genes targeted, we observed very pronounced and isolated heart phenotypes in *myrf* crispants (Fig. 2). We next assessed the myelin regulatory factor (*MYRF*) gene, encoding a transcription factor with an initially described critical role in central nervous system myelination (Emery et al., 2009). Although, to date, MYRF has been shown to directly regulate several genes implicated in oligodendrocyte differentiation and myelin formation (Bujalka et al., 2013), its gene expression domain indicates a broader role in various other tissues (Garnai et al., 2019; Hamanaka et al., 2019; Lonsdale et al., 2013; Qi et al., 2018). Recent studies have reported a new CUGS associated with *MYRF* mutations mapping to the conserved domains (Chitayat et al., 2018; Pinz et al., 2018; Qi et al., 2018; Rossetti et al., 2019; Tanaka et al., 2021). The *MYRF*-linked syndrome includes developmental anomalies of the heart (several structural defects), lung, diaphragm and urogenital tract (Rossetti et al., 2019). Targeting the myelin regulatory factor gene (*myrf*) led to substantial cardiac defects in 44% of the surviving injected embryos. We observed that 24% displayed a very robust and specific cardiac phenotype, including impaired cardiac looping, an elongated thin atrium and small ventricle, with a characteristic deflection at the atrioventricular connection and cardiac edema (further termed *myrf* mutant phenotype; Fig. 2; Fig. S3A). Finally, we reproduced the *myrf* mutant cardiac phenotype with two additional, independent sgRNA injections targeting *myrf* with a dual and triple sgRNA mix with rates of 54% and 61%, respectively (Fig. S3B). Given the distinctly identifiable and strong cardiac phenotype in *myrf* crispants, we focused on *myrf* as the most promising candidate for further analysis.

### Knockout of *myrf* results in specific heart defects in stable medaka mutants

**Fig. 3.**
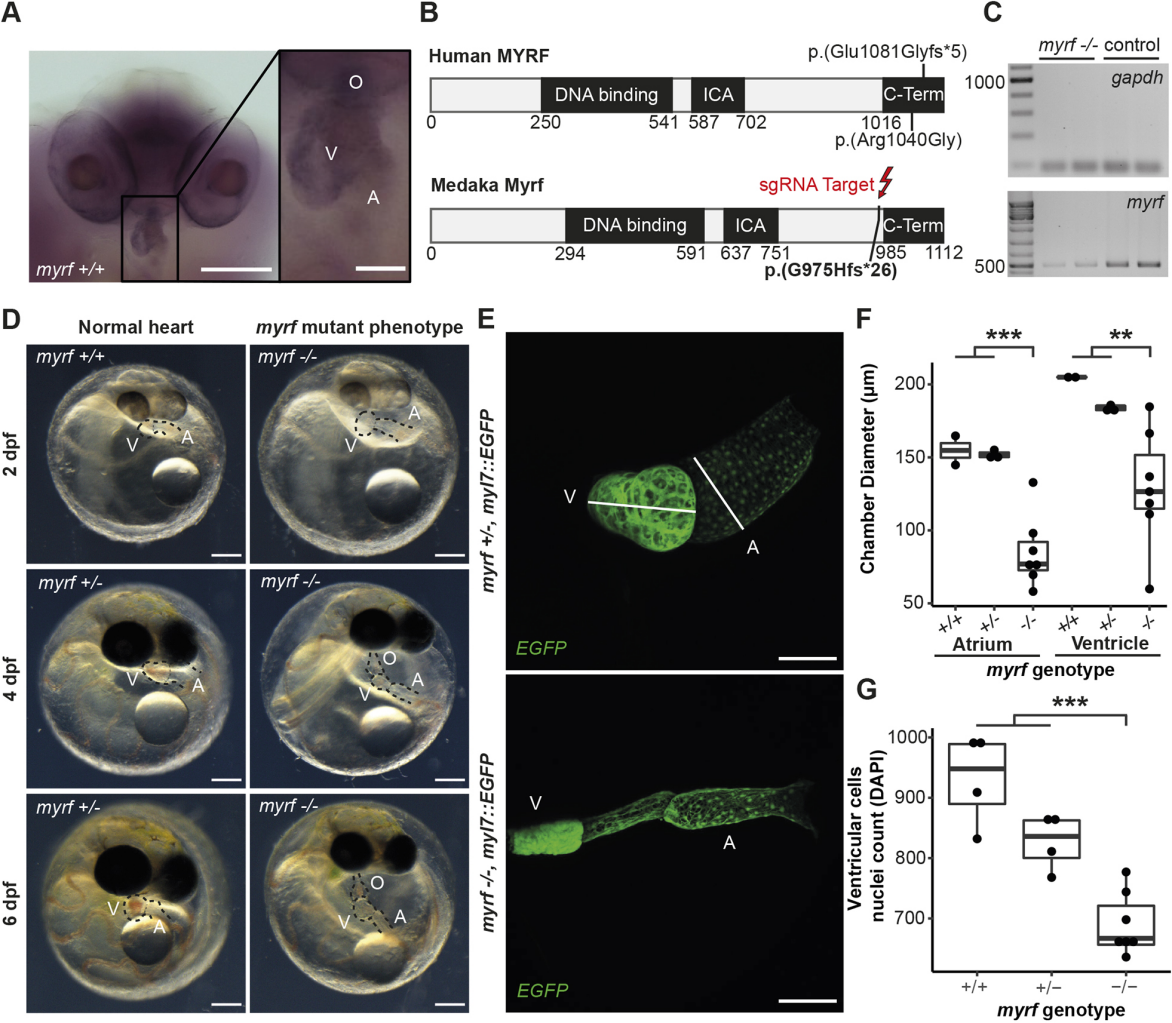
A *myrf* p.(G975Hfs*26) medaka mutant reveals progressive structural–functional heart defects. (A) *In situ* hybridization of *myrf* in medaka embryonic stage 36. Scale bars: 200 µm (left), 50 µm (zoom-in, right). (B) The human and medaka MYRF proteins feature conserved domains. The reported patient's mutation in MYRF C-terminal domain is indicated; the sgRNA target site is highlighted in the medaka Myrf. (C) Reverse transcription (RT)-PCR on *myrf* transcript in *myrf* mutant (two samples each from a pool of four embryos with characteristic *myrf*-related phenotypes) versus control (two samples each from a pool of four non-phenotypic siblings); positive control, GAPDH. (D) Time-series bright-field microscopy at 2, 4 and 6 dpf each for separate embryos derived from a medaka *myrf* p.(G975Hfs*26) incross shown for wild-type siblings (normal heart) and embryos displaying the *myrf* mutant phenotype. Individual genotyping yielded embryos with normal hearts, either *myrf*^+/+^ (embryo at 2 dpf) or *myrf*^+/−^ (embryos at 4 and 6 dpf); all phenotypic embryos had the genotype *myrf*^−/−^. Scale bars: 200 µm; bright-field images were equally adjusted for contrast in Fiji/ImageJ using the brightness and contrast function. (E) Whole-heart confocal microscopy of *myrf*^+/−^ and *myrf*^−/−^ embryos at 6 dpf, revealing heart phenotypes in homozygous mutants with looping defects in 6/6 specimens, intra-atrial constriction in 4/6 specimens, and 5/6 specimens with abnormal ventricle morphology; normal hearts were observed in 6/6 control siblings (all samples individually genotyped). Scale bars: 100 µm. (F) Quantification of atrial and ventricular diameters (axial in the ventricle, transverse diameter in the atrium as indicated in E at 6 dpf; *n*=2 (+/+), *n*=3 (+/−), *n*=7 (−/−). (G) Counts of cardiac ventricular cells in *myrf* heterozygous or homozygous mutants compared to wild-type siblings obtained from 11 planes with equal distance across entire ventricle volumes (DAPI-stained and confocal imaged); *n*=4 (+/+), *n*=4 (+/−), *n*=7 (−/−). Boxes represent the 25-75th percentiles, and the median is indicated; whiskers indicate minimum and maximum values within the first quartile (Q1) minus 1.5 times the interquartile range (IQR) and Q3 plus 1.5 times the IQR. Significance levels obtained using a two-tailed, unpaired, two-sample *t*-test (*myrf*^+/+^ and *myrf*^+/−^ pooled versus *myrf*^−/−^) (**P<0.005, ***P<0.0005). A, atrium; ICA, intramolecular chaperon autocleavage; O, outflow tract; V, ventricle.

To confirm our crispant *myrf* mutant phenotypes and characterize *myrf*-related pathologies in greater detail, we generated a stable *myrf* mutant line. *myrf* is expressed in the embryonic medaka heart, most prominently in the ventricle and outflow tract (at stage 36; Fig. 3A). We also detected cardiac *myrf* expression in the early tubular heart (stage 24) when the heart starts beating (Fig. S5A). Previous work showed that MYRF is localized in the endoplasmic reticulum (ER) and has three major and highly conserved protein domains: an N-terminal DNA-binding domain, an intramolecular chaperon autocleavage (ICA) domain and a C-terminal (ER lumen) domain (Fig. 3B). The ICA domain induces homotrimerization of the N-terminal fragment containing the DNA-binding domain. Autocleavage by the ICA domain releases the N-terminal DNA-binding homotrimer, which is transferred to the nucleus to exert transcriptional activity (Bujalka et al., 2013; Fan et al., 2021; Kim et al., 2017; Li et al., 2013). Interestingly, the regulative stimuli that trigger MYRF activation remain elusive.

As the transcription factor MYRF seems to control processes in heart development and has a high human relevance, we established a medaka *myrf* mutant model by outcrossing *myrf* crispants to wild-type fish. In one of the outcrosses, we recovered an exonic–intronic 182 bp deletion resulting in a frameshift-induced predicted premature termination codon (PTC) in exon 24 [p.(G975Hfs*26)]. This PTC is located next to the MYRF C-terminal domain, adjacent to two mutations detected in patients with hypoplastic left heart syndrome (HLHS) (Rossetti et al., 2019) (Fig. 3B; Fig. S4A,B). We established a *myrf* mutant line by outcrossing the corresponding founder with germline transmission of this 182 bp deletion to a wild-type *myl7::EGFP* reporter line (Gierten et al., 2020) to enable live imaging.

We next determined the effect of the generated PTC on *myrf* transcript levels by semi-quantitative reverse transcription (RT)-PCR. Mutant embryos show a marked reduction of *myrf* transcript, in contrast to wild-type siblings, indicative of degradation via nonsense-mediated mRNA decay (Fig. 3C). We reanalyzed the *myrf* mutant phenotype observed in crispants (F0) in non-mosaic embryos by incrossing heterozygous *myrf*^+/−^ fish. Homozygous p.(G975Hfs*26) mutant offspring recapitulated all features of the *myrf* mutant phenotype observed in the fraction (24%) of *myrf* crispants with the strongest expressivity (Fig. 3D-F). The *myrf* mutant phenotype, including cardiac edema, appeared early and was evident in bright-field microscopy at 2 dpf, when blood circulation and separation of the atrium and ventricle becomes apparent (stage 28; Iwamatsu, 2004), and showed progressive severity over time (Fig. 3D). An intra-atrial constriction was present in a subgroup of embryos (Fig. 3D,E). Both the constriction within the atrium visible in confocal microscopy (Fig. 3E) and the inflection at the atrioventricular connection potentially arise from an agglomeration of one side of the atrium and ventricle with the pericardial tissue (Fig. 3D,E; Fig. S5C). However, it would require a higher-resolution analysis of the relevant area to resolve this phenotype structurally.

Homozygous *myrf* mutants displayed a small ventricle, in which trabeculation was no longer discernible microscopically compared to the wild type (Fig. 3E,F). We next quantified the numbers of cardiac ventricular cells in homozygous *myrf* mutants. To this end, we used manual counting of 4′,6-diamidino-2-phenylindole (DAPI)-stained ventricular cell nuclei in 11 planes with equal distances across entire ventricle volumes as a proxy for overall ventricular cell number. Our quantification revealed a genotype-dependent decrease in ventricular cell numbers in *myrf* mutants, with intermediate numbers in heterozygous and minimal ventricular cells in homozygous embryos (Fig. 3G). Thus, mutated *myrf* leads to reduced ventricle size through decreased cell numbers (hypoplastic ventricle). All embryos with the *myrf* mutant phenotype were homozygous for the *myrf* p.(G975Hfs*26) allele (*n*=23) except one homozygous mutant with a normal heart (1/23), hinting at a generally strong, but occasionally incomplete, penetrance. In summary, the phenotype initially observed in *myrf* crispants was reproduced in a stable mutant line, which we propose as a new *in vivo* model to study the molecular mechanisms of the *myrf*-associated cardiac and extracardiac phenotypes.

### Modeling of human *MYRF* missense mutations in medaka with base editing causes embryonic heart defects

**Fig. 4.**
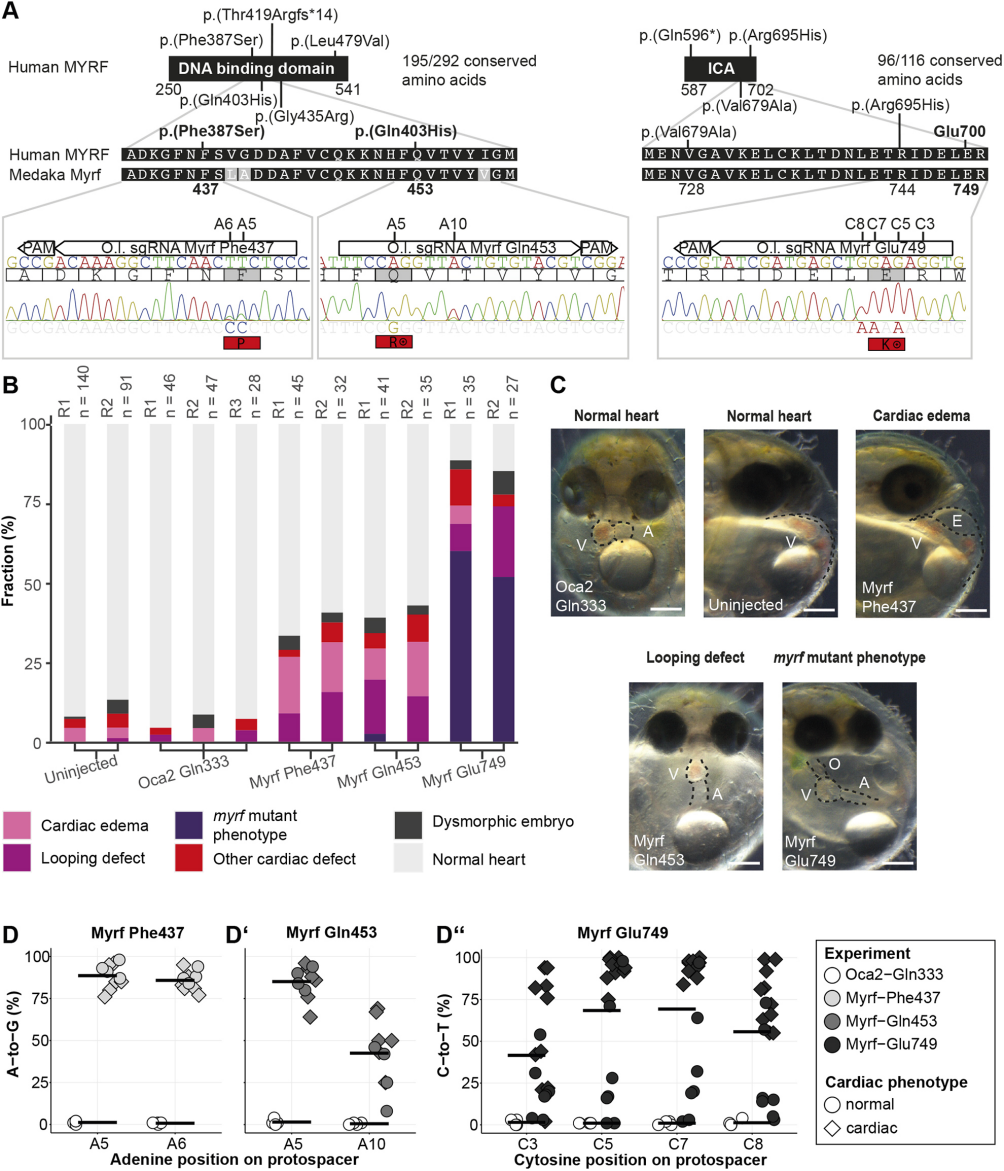
**Functional modeling of human missense mutations in MYRF using base editing in medaka induces embryonic cardiac defects.** (A) Location of known mutations in MYRF DNA-binding and ICA domains; protein alignment of corresponding protein segment between human and medaka indicates high conservation. Phe437 [orthologous to human p.(Phe387Ser)] and Gln453 [orthologous to human p.(Gln403His)] are directly targetable with adenine base editor (ABE); for human p.(Arg695His), a nearby orthologous amino acid to Glu700 (Glu749) was modified with a cytosine base editor (CBE). Corresponding sgRNA locations are given in the medaka *myrf* genomic sequence in the lower panel, with representative Sanger reads obtained from base-edited medaka embryos; note that Phe437 and Glu749 were edited via the non-coding DNA strand. Base numbering starts with one at the 5′ sgRNA end. (B) Proportion of cardiac phenotypes observed in surviving embryos of the indicated injections with two replicates per condition; examples for applied phenotype categories are displayed in C. ‘Other cardiac defects’ refers to additionally scorable defects [small ventricle, streaky heart, atrioventricular block, no blood flow or retrograde blood flow in the heart] according to the criteria defined in Figs S1 and S2; ‘*myrf* mutant phenotype’ refers to impaired cardiac looping, an elongated thin atrium, and small ventricle with a characteristic deflection at the atrioventricular connection and cardiac edema (see Fig. 3 and Fig. S3). (C) Bright-field images of 6 dpf embryos. Scale bars: 200 µm. Bright-field images were equally adjusted for contrast in Fiji/ImageJ using the brightness and contrast function, and were cropped from the original. (D-D″) Assessment of editing efficiencies in single medaka embryos edited at the sites of Myrf Phe437 (D), Gln453 (D′) and Glu749 (D″). Oca2 Gln333 editing group was used as negative control (no *myrf*-related genotypes or phenotypes). Sanger sequencing reads of individually genotyped embryos from experiments shown in B and C were analyzed with EditR tool. *n*=10 embryos for Phe437 and Gln453 (ABE8e), *n*=17 for Glu749 (evoBE4max), *n*=4 for each *oca2* control across all injections, which was injected with the CBE ancBE4max. A, atrium; E, edema; ICA, intramolecular chaperon autocleavage; O, outflow tract; PAM, protospacer adjacent motif; V, ventricle.

Interestingly, recent reports have associated MYRF mutations in patients with a newly described CUGS (Rossetti et al., 2019), including different types of CHD. We, therefore, sought to model genotype–phenotype correlations at the level of specific *myrf* point mutations in the functional domains of Myrf by DNA base editing in medaka (Fig. 4A). Recently developed and continuously optimized base editors enable direct generation of point mutations at targeted sites in genomic DNA (Anzalone et al., 2020; Huang et al., 2021). Base editors are engineered fusion proteins consisting of a functionally impaired Cas9 (usually a nickase) that locates a nucleobase deaminase sgRNA-guided to a genomic DNA sequence, allowing the deaminase to convert targeted DNA bases by hydrolytic deamination (Fig. 1B). Cytosine base editors (CBEs) technically enable cytosine to thymine (C-to-T) conversions, and adenine base editors (ABEs) facilitate adenine to guanine (A-to-G) editing; both leverage the endogenous repair and replication machineries (Gaudelli et al., 2017; Komor et al., 2016). We built on recent work that has characterized both ABE and CBE functionalities in medaka and has applied *in vivo* base editing to validate single-nucleotide variants (SNVs), demonstrating a high efficiency suitable for F0 phenotypic analysis (Cornean et al., 2022).

As human MYRF mutations associated with syndromic CHD cluster in two highly conserved MYRF domains, the DNA-binding and ICA domains, we asked whether missense mutations in these highly conserved protein domains are sufficient to induce developmental cardiac defects in the medaka model. In the DNA-binding domain of the transcription factor Myrf, we edited a nucleotide guided by the human missense mutation p.(Phe387Ser), which has been detected in a patient with aortic arch hypoplasia, coarctation of the aorta, HLHS and genitourinary anomalies (Qi et al., 2018). The orthologous phenylalanine (Phe437) in the medaka *myrf* is directly accessible for adenine base editing (Fig. 4A). Injection of sgRNA against Phe437 (DNA-binding domain) with ABE8e editor mRNA led to 29% and 38% surviving phenotypic embryos with heart phenotypes, including mainly cardiac edema and incomplete looping, in two replicates (Fig. 4B,C). However, the apparent specific heart defect observed in the *myrf* p.(G975Hfs*26) line was not evident in Phe437-injected samples (*n*=45) (Fig. 4B,C). Genotyping of individual ‘editants’ revealed A-to-G conversion rates at target adenines A5 and A6 ranging between 76-96% for this locus in phenotypic embryos. Embryos scored as phenotypically normal also gave high editing efficiencies (Fig. 4D), likely resulting from combined factors of variable penetrance of these mutations and individual mosaic compositions of edited cells differing between embryos. Among possible editing outcomes of the TTC codon (phenylalanine) within the window of maximal editing activity between nucleotides 4-8 (counted from the 5′ sgRNA site) are CCC (proline), CTC (leucine) and TCC (serine, the mutation found in the reported patient), of which phenylalanine-to-proline sequencing reads dominated.

To probe a second locus in the Myrf DNA-binding domain, we targeted glutamine 453 in medaka Myrf orthologous to the human mutation p.(Gln403His). This mutation has been detected in a patient with complex CHD (Scimitar syndrome, aortic arch hypoplasia, atrial septal defect, bicuspid aortic valve, HLHS, mitral stenosis, ventricular septal defect), cryptorchidism and diaphragm anomaly (Qi et al., 2018). In two replicates, injections of sgRNAs targeting the Gln453 locus with ABE8e base editor induced moderate cardiac aberrations of 34% and 40% in surviving injected embryos. Similar to the editing of the Phe437 locus, the predominant phenotypes were cardiac edema and looping defects. Editing efficiencies at the Gln403 orthologous locus at the relevant position A5 in medaka in phenotypic samples were in the range of 64-96%, changing the codons glutamine (CAG) to arginine (CGG) (Fig. 4D′). Further, we observed a bystander edit at position A10, changing the codon threonine (ACT) to alanine (GCT) with editing efficiencies between 25% and 69%. To score baseline phenotypes after base editing of a gene with no involvement in the heart, we used previously established base editing of *oca2*, an eye pigmentation-specific gene (Cornean et al., 2022). From this recent work, we used an sgRNA that creates a premature stop codon in *oca2* through cytosine editing (ancBE4max), resulting in predicted truncation at position Q333, in which embryos with cardiac edema and looping defects occurred at low frequencies comparable to those in the uninjected control group (Fig. 4B).

Next, we aimed to assess missense mutations in the ICA domain. *In silico* target site analysis identified a cytosine editing-accessible target, orthologous glutamate (749) to human Glu700, in proximity to human missense mutation p.(Arg695), which has been found in a patient with HLHS, genitourinary anomalies and congenital diaphragmatic hernia (Qi et al., 2018). Cytosine editing of the negatively charged Glu749 orthologous site in medaka (GAG) would result in positively charged lysine (AAG or AAA). We, therefore, selected the predicted Glu749Lys mutation potentially causing significant impairment to this enzymatically active domain to probe the functional impact of missense mutations in the Myrf ICA domain (Fig. 4A). Injection of an sgRNA designed to introduce Glu749Lys with the cytosine base editor evoBE4max yielded this missense mutation via C7 C-to-T editing at 84-100% efficiencies in phenotypic embryos (Fig. 4D″). Further, we observed inconsistent bystander editing (between 2% and 92%) outside the canonical editing window at C3, changing arginine (AGG) to lysine (AAG). Editing Glu749 led to phenotypic embryos in 89% and 78% of all surviving embryos, 60% and 52% of which displayed the *myrf* mutant cardiac phenotype in two replicates (Fig. 4B,C). We verified our results in F1 offspring from a *myrf* Glu749 editant incross, which yielded variable combinations of C3, C5, C7 and C8 C-to-T edited alleles. All analyzed embryos homozygous for the C7 C-to-T edit displayed the *myrf* mutant phenotype, and no embryo heterozygous or wild type for the edited C7 displayed the *myrf* mutant phenotype (Fig. S6).

**Fig. 5.**
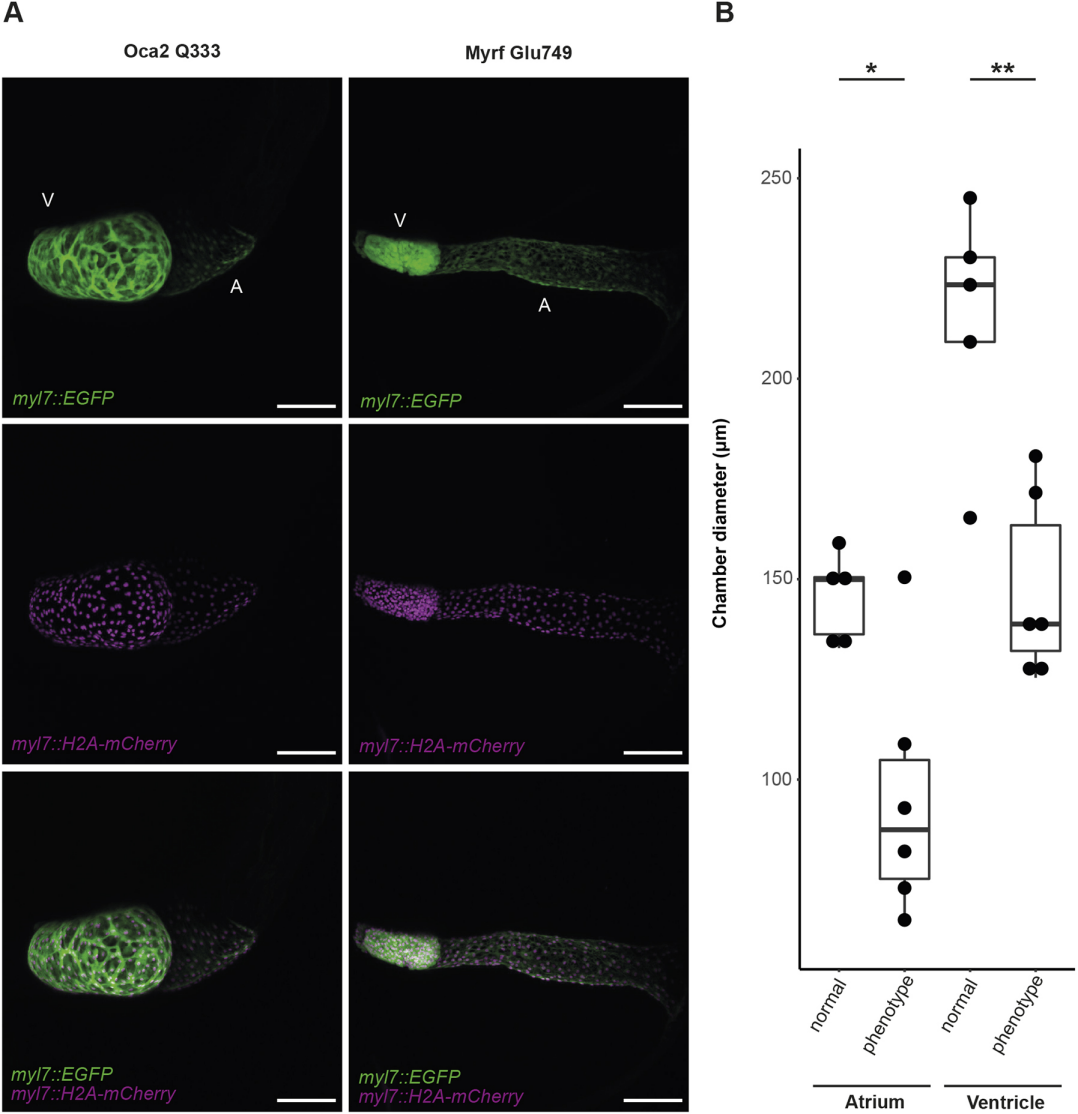
Myrf Glu749 base-edited embryos display cardiac defects similar to the myrf p.(G975H*fs) line phenotype. (A) Confocal imaging of Myrf Glu749 base-edited embryos. Myrf Glu749 sgRNA was co-injected with evoBE4max, *oca2* sgRNA was co-injected with ancBE4max into transgenic medaka *myl7::eGFP, myl7::H2A-mCherry* line. Imaging was performed at 6 dpf; *n*=5 (*oca2*, normal heart), *n*=6 (*myrf*, heart phenotype). Scale bars: 100 µm. (B) Quantification of atrial and ventricular diameters (axial in the ventricle, transverse diameter in the atrium, as indicated in Fig. 3E) at 6 dpf. Boxes represent the 25-75th percentiles, and the median is indicated; whiskers indicate minimum and maximum values within the first quartile (Q1) minus 1.5 times the IQR and Q3 plus 1.5 times the IQR. Significance levels obtained using a two-tailed, unpaired, two-sample *t*-test (*P<0.05, **P<0.005). A, atrium; V, ventricle.

Subsequently, Myrf Glu749 sgRNA and evoBE4max mRNA were injected into the *myl7::eGFP myl7::H2A-mCherry* medaka dual-color cardiac reporter line (Hammouda et al., 2021) to compare the phenotype in Glu749-edited embryos at cellular resolution with the heart phenotype of the *myrf* p.(G975Hfs*26) line. Confocal microscopy of hearts of edited embryos matched the phenotype of the *myrf* p.(G975Hfs*26) line, with impaired heart looping (6/6 embryos), tubular atrium and a constriction within the atrium in 3/6 embryos, and hypoplastic ventricle (5/6 embryos), compared to the normal heart in the *oca2* control (Fig. 5A,B).

In summary, functional interrogation of amino acids in medaka *myrf* equivalent or close to human *MYRF* missense mutations reported in patients with syndromic disease confirms the pathogenic role of these mutations. It shows that single missense mutations in highly conserved domains of Myrf can be sufficient to cause embryonic heart defects *in vivo*.

## DISCUSSION

Despite the high number of computationally identified genomic variants associated with CHD in large-scale sequencing studies, their functional simulation in model systems is currently a primary bottleneck. However, verifying putative causal variants is a prerequisite for translating genomic data to improve diagnosis and identification of novel therapeutic targets. Our phenotypic analysis of 12 preselected genes by CRISPR-Cas9-mediated targeting in medaka highlighted a novel cardiovascular phenotype spectrum of cardiac looping and morphological defects for five CHD-associated genes, *mapre2*, *smg7*, *cdc42bpab*, *myrf* and *ankrd11*, including a positive control *tbx1* (Table S2). Higher fractions of extracardiac phenotypes observed for *smg7* and *ankrd11* likely reflect these genes' broader function in multiple tissues. Our study pinpoints these genes as highly relevant targets for detailed mechanistic studies.

Given its highly penetrant and reproducible crispant phenotypes, we focused on the CHD gene *MYRF* that encodes a transcription factor. Initially associated with oligodendrocyte function (Emery et al., 2009), *MYRF* is widely expressed and has recently been associated with CHD (Garnai et al., 2019; Hamanaka et al., 2019; Homsy et al., 2015; Jin et al., 2017; Lonsdale et al., 2013; Qi et al., 2018). It is highly expressed in embryonic mouse heart (among the top 14% of genes) and has high probability of loss-of-function intolerance (pLI of 1) (Homsy et al., 2015; Jin et al., 2017). The initial descriptions of *MYRF*-related syndromic disease (Chitayat et al., 2018; Pinz et al., 2018) led to a new definition of a rare cardiac-urogenital syndrome [CUGS; Online Mendelian Inheritance in Man (OMIM) #618280] that includes CHD, genitourinary anomalies, congenital diaphragmatic hernia and pulmonary hypoplasia (Chitayat et al., 2018; Krane et al., 2021; Qi et al., 2018; Rossetti et al., 2019; Tanaka et al., 2021). Although the phenotypic presentation associated with *MYRF* mutations varies, CHD is the most common, of which primarily reported cardiac phenotypes are HLHS and scimitar syndrome (summarized in Rossetti et al., 2019). Other heart defects include tetralogy of Fallot, septal defects, valvular defects, hypoplastic aortic arch and patent ductus arteriosus.

The MYRF-associated human heart defects suggest that this transcription factor plays a central role in heart development and function, but to our knowledge has so far only been studied experimentally in the context of increased heartbeat (Hammouda et al., 2021). We, therefore, targeted *myrf* in medaka embryos, which resulted in a characteristic and highly penetrant heart phenotype, including aberrant heart looping, impaired atrioventricular maturation, and hypoplastic ventricle with cardiac edema, visible already at early developmental stages.

Interestingly, whereas in *MYRF*-related heart disease, HLHS is the leading phenotype, we observed hypoplastic ventricle and thin and elongated atrium upon targeted inactivation of *myrf*. Given the recently reported frameshift MYRF variants found by exome sequencing of 87 HLHS parent–offspring trios (Krane et al., 2021), our genotype–phenotype data support the pathogenic relevance of MYRF within the complex genetics of HLHS. Our data show nonsense-mediated mRNA decay as a pathogenic mechanism leading to cardiac malformation in medaka *myrf* mutants. Additionally, the lack of interaction between MYRF and TMEM98, an ER-associated transmembrane protein, inhibits its autocleavage via the MYRF C-terminus (Huang et al., 2021), which might be another factor contributing to the phenotype.

By contrast, little is known about the *cis*-regulatory elements bound by MYRF responsible for the prominent heart phenotype. RNA-sequencing (RNA-seq)-based differential gene expression analysis revealed the transcription factor *GATA4* as one possible downstream effector of MYRF (Bujalka et al., 2013), corroborated by a substantial overlap between our *MYRF* mutant phenotypes and *GATA4* zebrafish morphants, including defective heart looping, failure of chamber expansion and cardiac edema (Holtzinger and Evans, 2005). While the complete set of cardiac-relevant MYRF target genes remains unknown, our medaka *myrf* mutant line constitutes an excellent resource for tissue-specific delineation of Myrf downstream actors by chromatin immunoprecipitation with sequencing (ChIP-seq), assay for transposase-accessible chromatin with sequencing (ATAC-seq) and RNA-seq data.

The mosaic nature of Cas9-based targeted gene inactivation and allele heterogeneity in F0 can spread the phenotypic spectrum and limit the certainty of phenotype evaluation in the injected generation. However, in the case of *myrf*, the matching phenotype profiles in crispants and homozygous mutant embryos highlight the potential of CRISPR-Cas9-based F0 approaches for initial phenotypic assessment based on the high efficiency of an optimized Cas9 variant applied in medaka to generate biallelic mutations (Hammouda et al., 2021; Thumberger et al., 2022). Also, targeting the positive control gene *tbx1* yielded a highly penetrant phenotype encompassing looping defect, ventricular malformation and oropharyngeal malformation, in line with principle phenotypic features in zebrafish, and mirroring cardiac and facial DiGeorge syndrome features linked to *TBX1* in human (Choudhry and Trede, 2013; Jerome and Papaioannou, 2001; Zhang et al., 2006). Our findings show that F0 phenotypes can recapitulate homozygous mutant phenotypes in line with accumulating evidence in fish models (Ablain et al., 2015; Burger et al., 2016; Cornean et al., 2022; Hammouda et al., 2021; Jao et al., 2013; Shah et al., 2015; Wu et al., 2018).

Our analyses uncovered a substantial cardiac involvement of *myrf* in medaka. Our subsequent assessment of specific *MYRF* mutations associated with syndromic disease, including congenital heart defects, took advantage of the high editing efficiencies in medaka using the evoBE4max and ABE8e base editors, precisely inducing nucleotide changes at desired positions in contrast to random indel formation by Cas9 (Cornean et al., 2022). We introduced mutations in the ICA or DNA-binding domain to most directly interfere with the transcriptional activity as suggested by the clustering of most CUGS-associated mutations in these domains. The creation of Phe437Pro and Gln453Arg missense mutations in the DNA-binding domain in medaka *myrf* [orthologous to human MYRF p.(Phe387Ser) and p.(Gln403His) loci, identified in syndromic patients with CHD (Qi et al., 2018)], using ABE8e, induced moderate proportions of cardiac phenotypes in F0-edited embryos, including abnormal cardiac looping and primarily heart edema, confirming the clinical relevance of missense mutations in the MYRF DNA-binding domain *in vivo*. We noted a comparatively mild phenotypic penetrance and expressivity for mutations in the DNA-binding domain compared to a mutation created in the ICA domain. We speculate that the induced missense mutations in the Myrf DNA-binding domain generated hypomorphic alleles resulting in different binding affinities to certain, tissue-specific *cis*-regulatory elements. In contrast, mutations in the ICA-domain p.(Val679Ala) and p.(Arg695His) interfere with autocleavage of MYRF and with the structure of MYRF trimers. This results in the complete loss of transcriptional activity of these protein variants, likely via haploinsufficiency (An et al., 2020). Given the ICA domain's clinical relevance, we studied this domain *in vivo* and created a missense mutation at the orthologous site of the human Glu700, including a charge switch (Myrf p.Glu749Lys). We observed highly penetrant heart phenotypes matching the pronounced phenotype seen in *myrf* p.(G975Hfs*26) mutants, compatible with the hypothesized more severe effect of the side chain switched charge in this enzymatically active, and functionally indispensable, domain (An et al., 2020). Notably, editing of the orthologous amino acid to MYRF Glu700 (medaka Glu749), closely located to p.(Arg695His) associated with HLHS (Rossetti et al., 2019), led to a hypoplastic heart phenotype in medaka.

We present a compelling *in vivo* modeling of single *MYRF* mutations by directly targeting specific amino acids with base editing. Our approach demonstrates that patient-based *MYRF* mutations cause complex heart phenotypes in medaka, highlighting the clinical significance of single missense mutations. Stable lines harboring intended point mutations will help to disentangle the exact phenotypic consequences of each missense mutation.

There are a few notable differences between the human MYRF pathology and the medaka *myrf* model. Whereas CUGS appears to be mainly caused by haploinsufficiency, we observed the phenotype in a homozygous medaka mutant. Heterozygotes presented normal cardiovascular development in bright-field microscopy. However, detailed analysis in confocal microscopy revealed reduced cardiac ventricular cell number and slightly reduced ventricle size. The hypoplastic heart phenotype of homozygous offspring of the medaka *myrf* p.(G975Hfs*26) line represents the cardinal phenotype of human *MYRF*-related syndromic disease. However, we have not investigated central nervous system (CNS) myelination, which, during the course of this study, has been found to be significantly decreased in a zebrafish *myrf* mutant with behavioral and electrophysiological consequences (Madden et al., 2021). Therefore, our medaka *myrf* mutant line will be instrumental as a complementary model to further study the CNS functions of Myrf.

In summary, the sequential process of *in silico* prioritization combined with CRISPR-Cas9 targeted mutagenesis highlighted the significance in heart development of several novel CHD candidate genes, paving the way for further mechanistic studies in mutant lines. Clinically, the newly determined causality for *MYRF* contributes to refining genetic diagnoses, specifying the prognosis of individual gene variants and uncovering disease mechanisms for new therapy development. The efficiency and scalability of base editing to model specific point mutations accelerate the generation of human-relevant disease models.

## MATERIALS AND METHODS

### CHD gene selection and prioritization

Thirty recent publications (2015-2020) on large-scale sequencing or single-gene studies on CHD were selected for significant genotype–phenotype associations. Candidate gene enumerations of these publications were included in the search space of this study until a target number of 200 candidate genes was reached (Bravo-Gil et al., 2019; Cohen et al., 2020; Dasouki et al., 2020; Demal et al., 2019; Dianatpour et al., 2020; Fotiou et al., 2019; Hawer et al., 2020; Hay et al., 2020; Haynes et al., 2020; Homsy et al., 2015; Ji et al., 2020; Jin et al., 2017; Lee et al., 2020; Liu et al., 2017, 2020; Mastromoro et al., 2020; Morton et al., 2020; Muir et al., 2020; Page et al., 2019; Priest et al., 2016; Reuter et al., 2020; Richter et al., 2020a; Samudrala et al., 2020; Sifrim et al., 2016; Szot et al., 2018, 2019; Wang et al., 2020a,b; Xu et al., 2018). The Ensembl and PubMed databases were searched for previous work detailing structural–developmental cardiac defects *in vivo* upon knockout or knockdown of the candidate gene. For 66 genes, no such data were identified, and at least one medaka ortholog to the human gene was annotated in Ensembl. Subsequently, evolutionary conservation from human to medaka fish of these genes was assessed using Ensembl orthology scores. Manual alignment of amino acid sequences in select cases was performed to assess the conservation of protein domains harboring the patient mutation. To avoid confounding by genetic compensation, candidates with two highly conserved orthologs were excluded. From the remaining 37 genes, promising candidates were identified by individual prioritization assessing the amount of associated CHD cases and prior *in vitro* studies that imply relevance for cardiac structural development. Genes were ranked higher if cases of isolated CHD were reported, resulting in a final selection of 12 genes that entered the experimental phase. See Tables S1 and S2 for a summary of all genes and the filtering steps.

### Fish maintenance

Medaka (*Oryzias latipes*) stocks were maintained (fish husbandry, permit number 35-9185.64/BH Wittbrodt), and experiments (permit number 35-9185.81/G-271/20 Wittbrodt) were performed following local animal welfare standards (Tierschutzgesetz §11, Abs. 1, Nr. 1) and European Union animal welfare guidelines (Bert et al., 2016). Fish were maintained in closed stocks and constant recirculating systems at 28°C on a 14 h light/10 h dark cycle. The fish facility is under the supervision of the local representative of the animal welfare agency. The following medaka lines were used: Cab as wild type (Loosli et al., 2000), Cab (*myl7::EGFP*) (Gierten et al., 2020), HdrR (*myl7::EGFP, myl7::H2A-mCherry*) (Hammouda et al., 2021), Cab (Myrf p.(G975Hfs*26) (this work).

### Plasmids and mRNA

Cas9 mRNA was cloned according to a previously detailed protocol (Meyer et al., 2020). ancBE4max, evoBE4max and ABE8e mRNA were synthesized following previously published protocols (Cornean et al., 2022).

### sgRNA design and synthesis

sgRNAs were designed using Geneious software (Biomatters) in conjunction with medaka and human genome data acquired from the Ensembl database (Ensemble release 101; August 2020). For Cas9 experiments, an off-target prediction was performed utilizing the CCTop algorithm (Stemmer et al., 2015); sgRNAs were cloned as described previously (Stemmer et al., 2015). A second set of sgRNAs of Myrf Phe437, Gln453 and Glu749 was ordered via the Integrated DNA Technologies (IDT) synthesis service (Table S3).

### Microinjections

Microinjection into fertilized one-cell-stage medaka zygotes was performed as described previously (Rembold et al., 2006). For each replicate in the F0 screen (Fig. 2), 70 specimens were injected; separate Oca2 controls, each with 30 injected specimens, were generated with each gene injection. For Cas9 experiments, two sgRNAs targeting the candidate gene were injected at a concentration of 15 ng/µl per sgRNA with 150 ng/µl Cas9 mRNA and 10 ng/µl GFP mRNA. Myrf was targeted with one sgRNA that was previously validated (Hammouda et al., 2021). For the *oca2* control, 30 ng/µl *oca2* sgRNA T3 was injected. The functionality of sgRNAs was validated by identifying editing events in genotyping a pool of ten embryos derived from one of the replicates.

For base-editing experiments, 30 ng/µl sgRNA, 150 ng/µl base editor mRNA and 10 ng/µl GFP mRNA were used. Injection mixes were prepared in RNAse-free H_2_O. After injection, embryos were placed in ERM (17 mM NaCl, 40 mM KCl, 0.27 mM CaCl_2_, 0.66 mM MgSO_4_, 17 mM Hepes). Embryos were screened for homogeneous GFP expression at 5 h post fertilization, and GFP-negative embryos were discarded. Subsequently, embryos were placed in a medaka hatch medium [2 mg/l Methylene Blue trihydrate (Sigma-Aldrich) in 1× ERM] and incubated at 28°C. At 4 and 6 dpf, embryos were screened for cardiovascular phenotypes using a set of standardized criteria displayed in Fig. 2, and Figs S1 and S2. All embryos with cardiovascular phenotypes were recorded with a 5 s bright-field microscopy video. Extracardiac manifestations scoring (Fig. 2; Figs S1 and S2) and scoring for the *myrf* mutant phenotype (Fig. S3) were performed using these video recordings. Scoring categories for extracardiac manifestations (medium, strong; Fig. S1) were subsumed in ‘cardiac+extracardiac phenotype’ (medium+strong) in Fig. 2A. All surviving embryos were included in the analysis (=*n*). The investigators were aware of the allocated experimental group.

### Genotyping, genome-editing validation and quantification

Single embryos or pools of ten were placed in 100 and 200 µl DNA extraction buffer (100 mM Tris-HCl pH 8.5, 10 mM EDTA pH 8, 200 mM NaCl, 2% sodium dodecyl sulfate) with 5 or 10 µl proteinase K (20 mg/µl), respectively, and incubated at 60°C overnight. Proteinase K was inactivated at 95°C for 20 min, and the solution was diluted to 200 or 400 µl, respectively, with RNase-free water. Fin clip samples for genotyping of adult fish were put in 100 µl 50 mM NaOH and incubated at 95°C for 15 min. Then, 25 µl 50 mM Tris-HCl pH 8 was added. For genotyping PCR, a 50 µl (in H_2_O) template reaction of 1× Q5 reaction buffer and 0.3 µl Q5 polymerase (2000 U/µl) (NEB), 1 µl dNTPs (10 mM), 2 µl forward primer (10 µM), 2 µl reverse primer (10 µM) and 1 µl DNA sample was used. The following thermocycler settings were used: initial denaturation at 98°C for 2 min, denaturation at 98°C for 30 s, annealing temperature calculated using the TM calculator tool (NEB) for 30 s, extension at 72°C for an extension time calculated based on an amplification speed of 1 kb per 30 s of the DNA polymerase, final extension at 72°C for 5 min, and cool down at 10°C for 1 min. Slight adjustments were made for individual primer pairs if PCR failed. Following agarose gel electrophoresis, the bands were purified with an InnuPrep PCR pure kit (Analytik Jena) or a Monarch DNA gel extraction kit (NEB). Sanger sequencing was performed through the Eurofins genomics sequencing service (see Table S4 for primer sequences).

The editing events (control and sgRNA of interest) were validated by aligning sequencing reads to the reference genome using Geneious software (Biomatters). Sanger sequencing reads were analyzed with the online TIDE tool (http://tide.nki.nl; Brinkman et al., 2014) to estimate editing efficiencies of sgRNAs used in CRISPR-Cas9-based screening of CHD candidate genes (Table S3). Base editing efficiencies were calculated with the online EditR tool (http://baseeditr.com; Kluesner et al., 2018).

### Medaka *myrf* p.(G975Hfs*26) mutant line generation

Crispants of *myrf* T1 injection were grown to adulthood and outcrossed to a Cab cardiac reporter line *myl7::EGFP* (Gierten et al., 2020). Genotyping of the offspring revealed one female transmitting the *myrf* p.(G975Hfs*26) allele. These F1 offspring were raised to adulthood and genotyped as described. Heterozygous F1 fish carrying the *myrf* p.(G975Hfs*26) allele were incrossed to obtain homozygous F2 mutants. As homozygous adults develop phenotypes, the line is kept as heterozygotes, which are not phenotypic. Crosses of the subsequent generation (F3) reproduced the findings described in Fig. 3D and were used for the DAPI staining of *myrf* mutant ventricles (Fig. 3G).

### RT-PCR on *myrf* p.(G975Hfs*26) mutant embryos

Total RNA was extracted from two pools of four embryos with *myrf* mutant phenotype and two pools of four wild-type sibling embryos using Trizol (Thermo Fisher Scientific) according to the manufacturer's protocol except the following modifications: 1 ml Trizol was used before the precipitation and additional chloroform purification was performed; thereafter, isopropranolol purification (2.5× volume of chloroform) was performed with 1 µl glycogen (1 mg/ml, RNase-free) to 500 µl isopropranolol. Reverse transcription was performed with a RevertAid Kit (Thermo Fisher Scientific) according to the standard protocol using oligo(dT)18 primers. The input total RNA for each sample reaction was 500 ng. Each cDNA sample was diluted 1:1 with nuclease-free H_2_O. 25 µl PCR mixes (in H_2_O) included the following: 1× Q5 reaction, 0.5 µl dNTPs (10 mM), each 1 µl forward and reverse primer (10 µM), 0.2 µl Q5 polymerase (2000 U/ml, NEB) and 1 µl cDNA. The following thermocycler settings were used: initial denaturation at 98°C for 2 min, denaturation at 98°C for 30 s, annealing temperature of 66°C (myrf_p(G975HfsStop26)_F1/R1), 67°C (myrf_p(G975HfsStop26)_F2/R2) and 64°C (GAPDH_F1/R2), extension at 72°C for 12 s (myrf_p(G975HfsStop26)_F1/R1), 35 s (myrf_p(G975HfsStop26)_F2/R″) and 10 s (GAPDH_F1/R2), final extension at 72°C for 5 min, and cool down at 12°C for 10 min. Primer sequences were as follows: myrf_p(G975HfsStop26)_F1, 5′-TCACAAGTAGCGTTTGGGCA-3′; myrf_p(G975HfsStop26)_R1, 5′-GAAATCCAAGAGCGTTGATCTGT-3′; myrf_p(G975HfsStop26)_F2, 5′-TCGGTGCCTGTGTTGTCTTT-3′; myrf_p(G975HfsStop26)_R2, 5′-TGATCACTGCCTTTCTGAGCA-3′; GAPDH F1, 5′-AAAGTCATTCACGATAACTTTGGCA-3′; GAPDH R2, 5′-TAGGACCATCCACTGTCTTCTGAG-3′.

### Bright-field and confocal imaging of whole embryos

Bright-field images were acquired with a Nikon SMZ18 fitted with a Nikon DS-Fi2 camera using Nikon NIS-Elements software. For confocal imaging, embryos were incubated in ERM at 28°C, and injections were administered to the HdrR *myl7::EGFP, myl7::H2A-mCherry* reporter line (Hammouda et al., 2021). The *ankrd11* T1+T2- and *tbx1* T1+T2-injected embryos with the respective *oca2* control were imaged ventrally and treated with 5× N-phenylthiourea (PTU)/1× ERM (50× PTU: 0.33 g PTU in 200 ml H_2_O, Sigma Aldrich) at 4 dpf. The *myrf* T1-injected, Myrf Glu749-injected and myrf p.(G975Hfs*26) embryos were imaged laterally. At 6 dpf, embryos were treated with 200 mg/l tricaine (ethyl 3-aminobenzoate methanesulfonate, Sigma Aldrich) and 50 mM 2,3-butanedione 2-monoxime (BDM; Abcam) for 40-60 min to stop cardiac contraction. Subsequently, embryos were transferred to a glass-bottom dish with micro-well cover glass (MatTek) containing a mounting solution consisting of 1% low-melting agarose, 50 mM BDM and 200 mg/l tricaine. Specimens were imaged on an SP8 confocal microscope (Leica) with a 20× objective (glycerol). Fig. 3F and Fig. 5B measurements were taken at the largest ventricular and atrial diameters, as displayed in Fig. 3E, and all acquired confocal images were included in the analysis (*n*).

### DAPI staining, imaging and cell number quantification in *myrf* mutant heart (ventricles)

Embryos were collected from *myrf* p.(G975Hfs*26) incrosses and fixed at 6 dpf in 4% paraformaldehyde in PTW [PBS (Thermo Fisher Scientific) with 0.05% Tween 20] and washed in PTW four times. The genotyping protocol was adjusted to small volumes: the embryos tails were separated from the trunk and incubated in 50 µl DNA extraction buffer and 2 µl proteinase K (20 mg/ml) at 60°C overnight; then, 100 µl H_2_0 was added, and samples were heated for 15 min at 95°C. The imaging samples (head to trunk, including the heart) were stained with 5 µg/ml DAPI nuclear staining agent and 1% dimethyl sulfoxide (DMSO) in PTW for 15 min and subsequently washed in PTW three times. Subsequently, whole hearts were dissected from the trunk and yolk, mounted in 20% urea, 30% D-sorbitol, 5% glycerol in DMSO and imaged with a SP8 confocal microscope (Leica) with a 63× objective. Acquired confocal stacks were divided into 11 planes of equal distance, in which nuclei were counted manually using Fiji’s ‘cell counter’ function [total *n*=15; by genotyping, *n*=4 (*myrf*^+/+^), *n*=4 (*myrf*^+/−^), *n*=7 (*myrf*^−/−^)].

### *In situ* hybridization

Whole-mount *in situ* hybridization using NBT/BCIP detection was carried out as previously described (Loosli et al., 1998). Samples were mounted in 86% glycerol imaged in bright-field microscopy (SMZ18, Nikon). The *in situ* probe was derived from an in-house clone library, clone P36J_24, a 2.7 kb fragment covering *myrf* C-terminal exons and untranslated region hold in a pCMVSport6.1 vector, which was transcribed with T7 polymerase.

### Data analysis and statistics

F0 Cas9 screen test gene replicates included each one *oca2* control. Individual replicates of the candidate genes were tested with a Fisher's exact test against the number of cardiac phenotypes in the pooled *oca2* control injections. If one of two replicates did not reach the significance threshold, the gene's effect was rated as insignificant. Statistics were calculated in R (http://www.r-project.org). Ensembl ID ENSG00000124920 (*MYRF*) and ENSORLG00000006459 (*myrf*) were used to generate Fig. 3A, Fig. 4A and Fig. S4.

### Data visualization

Maximum projections of confocal images were generated in Fiji/ImageJ (Schindelin et al., 2012). Individual frames from bright-field videos were captured and adjusted for contrast (same settings for control and experimental group) in Fiji/ImageJ using the brightness and contrast function (Figs 2-5; Figs S1, S3 and S6).). Bright-field images from Figs 2 and 4 were cropped from the original. Parts of Fig. 1 were created with elements from BioRender.com. Figure graphs were generated with ggplot2 (Wickham, 2016) and edited aesthetically in Adobe Illustrator.

## Supplementary Material

10.1242/dmm.049811_sup1 Supplementary information
