# Supplementary material for: CRISPR-based knockout and base editing confirm the role of MYRF in heart development and congenital heart disease
Source: Dis Model Mech. 2023 Aug 16;16(8):dmm049811. doi: 10.1242/dmm.049811 (PMC10445736; doi:10.1242/dmm.049811)
Supplement: Supplementary information [file dmm-16-049811-s1.pdf]

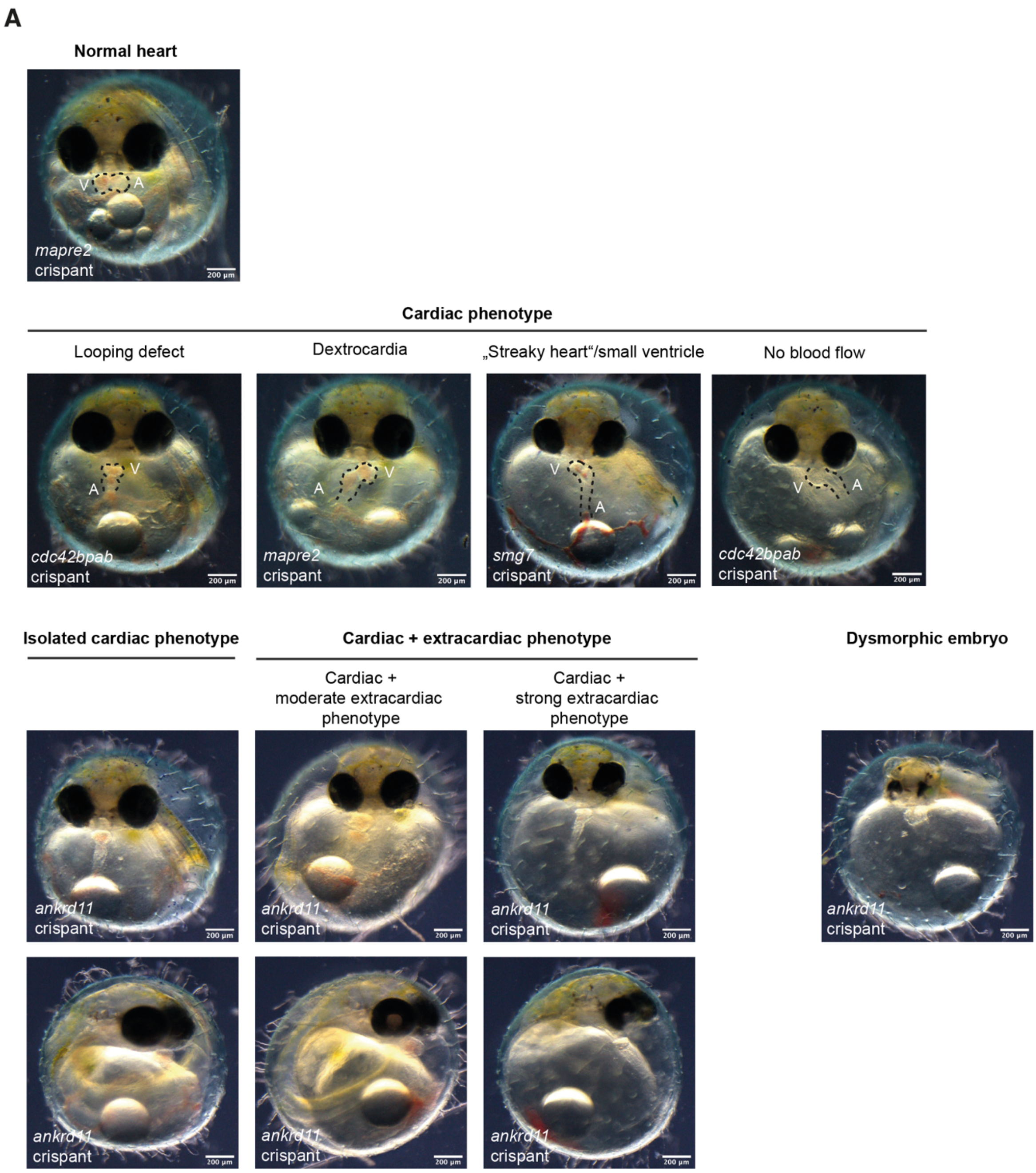

Disease Models & Mechanisms • Supplementary information

Fig. S1. Phenotype classification criteria used in the CRISPR-Cas9 screen. Cardiac phenotypes were identified based on standardized phenotypic features: Looping defect, dextrocardia, small ventricle, streaky heart, AV block, no blood flow or retrograde blood flow in the heart. Further, based on extracardiac manifestations, embryos were classified into three groups: isolated cardiac (no discernable extracardiac malformation), cardiac and extracardiac phenotype (heart malformation and moderately or strongly affected extracardiac organs), and dysmorphic embryo (whole embryo severely malformed). Bright-field whole-embryo images illustrate each phenotype group; these screening criteria were applied in Fig. 2A and Fig. S2A-B; to enhance the readability of Fig. 2A, “Cardiac + moderate extracardiac phenotype” and “Cardiac + strong extracardiac phenotype” were subsumed into one group “Cardiac + extracardiac phenotype”. Bright-field images illustrating the screening criteria utilized in Fig. 2A and Fig. S2A-B on whole embryos; images were recorded at 6 dpf and equally adjusted for contrast in Fiji/ImageJ using the brightness and contrast function; 6 dpf *cdc42bpab*, *smg7*, *mapre2* crispant bright-field images are displayed in a cropped version in Fig. 2B; A: atrium, V: ventricle.

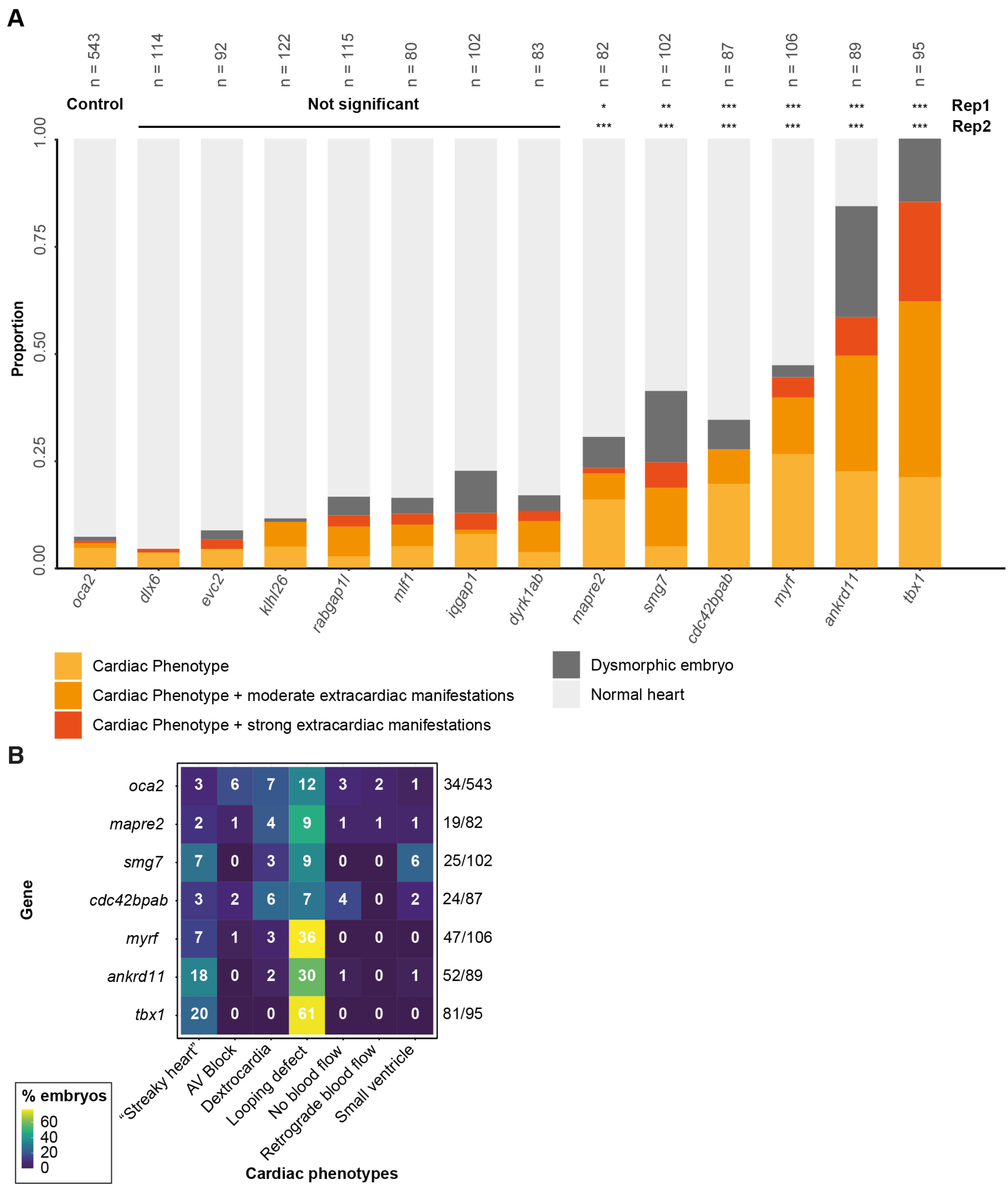

Fig. S2. Screening results for 12 CHD-associated genes of F0 CRISPR-Cas9-based targeting. (**A**) Summary of observed phenotypes at 6 dpf for each targeted gene (2 pooled replicates). Oca2 was a negative control, setting the baseline for injection procedure-induced phenotypes (pooled control injections, see Methods). Five out of 12 CHD genes displayed significant cardiac phenotypes in crispants. Tbx1 with known heart and extracardiac functions served as a positive control to capture and discriminate cardiac and extracardiac phenotypes in F0 CRISPR-Cas9-based gene targeting. Statistics given per replicate vs. *oca2* control as a Fisher's exact test; significance levels are *p<0.05, **p<0.005, ***p<0.0005. In Fig. 2A, scoring categories for extracardiac manifestations (moderate, strong; Fig. S1) were summarized in "cardiac- + extracardiac phenotype". (**B**) Heatmap representation of embryos with cardiac phenotypes from (A) resolved by cardiac phenotype categories (absolute numbers), ratios indicate embryos with cardiac phenotype/all injected embryos.

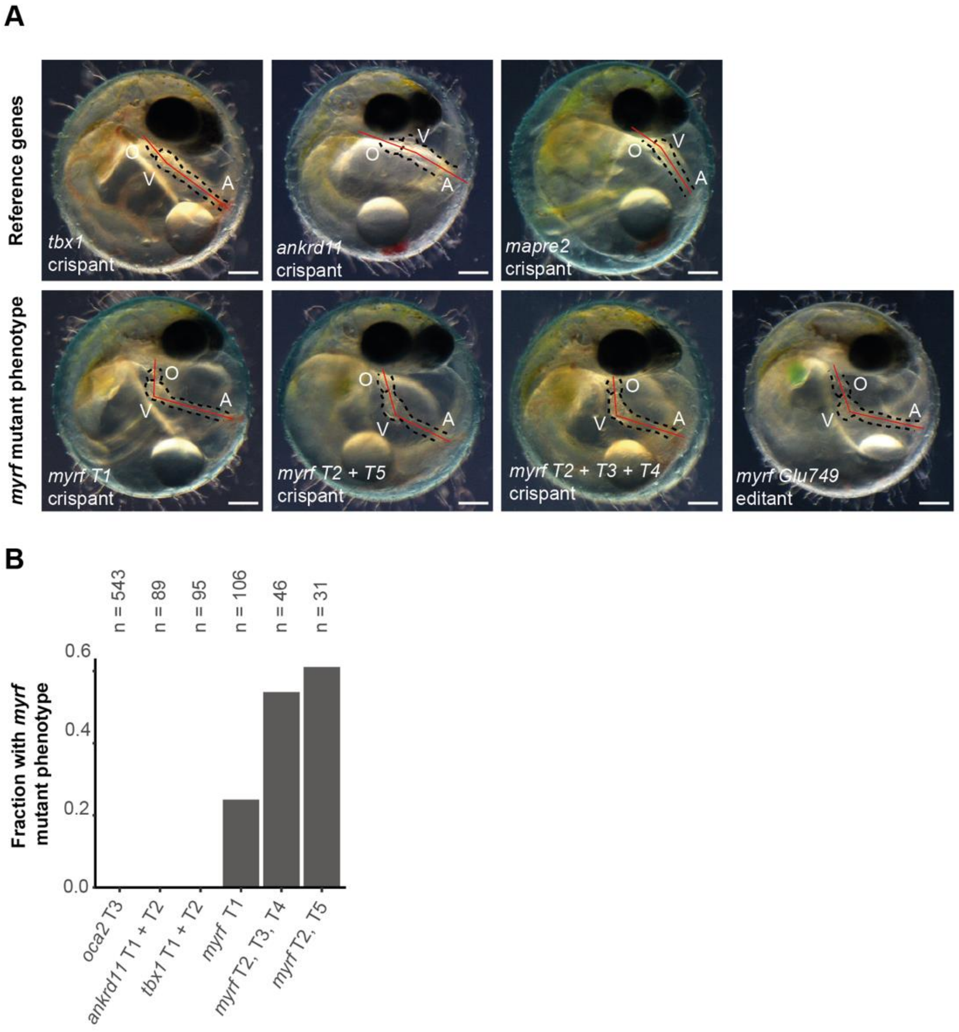

Fig. S3. *myrf* mutant phenotype. (**A**) Characteristic heart phenotype of *myrf* crispants and editant (6 dpf) shown in lateral view highlighting the typical deflection at the atrioventricular connection compared to looping defects observed for other target (reference) genes; dashed line indicates the heart circumference, red line indicates the angle between atrium and outflow tract; A: atrium, V: ventricle, O: outflow tract, bright-field images were equally adjusted for contrast in Fiji/ImageJ using the brightness and contrast function, scale bar: 200 µm. (**B**) Frequencies of *myrf* mutant phenotype detected after targeting *oca2* (one sgRNA), *ankrd11* (two sgRNAs), *tbx1* (two sgRNAs), and *myrf* (combinations of five different sgRNAs).

Disease Models & Mechanisms • Supplementary information

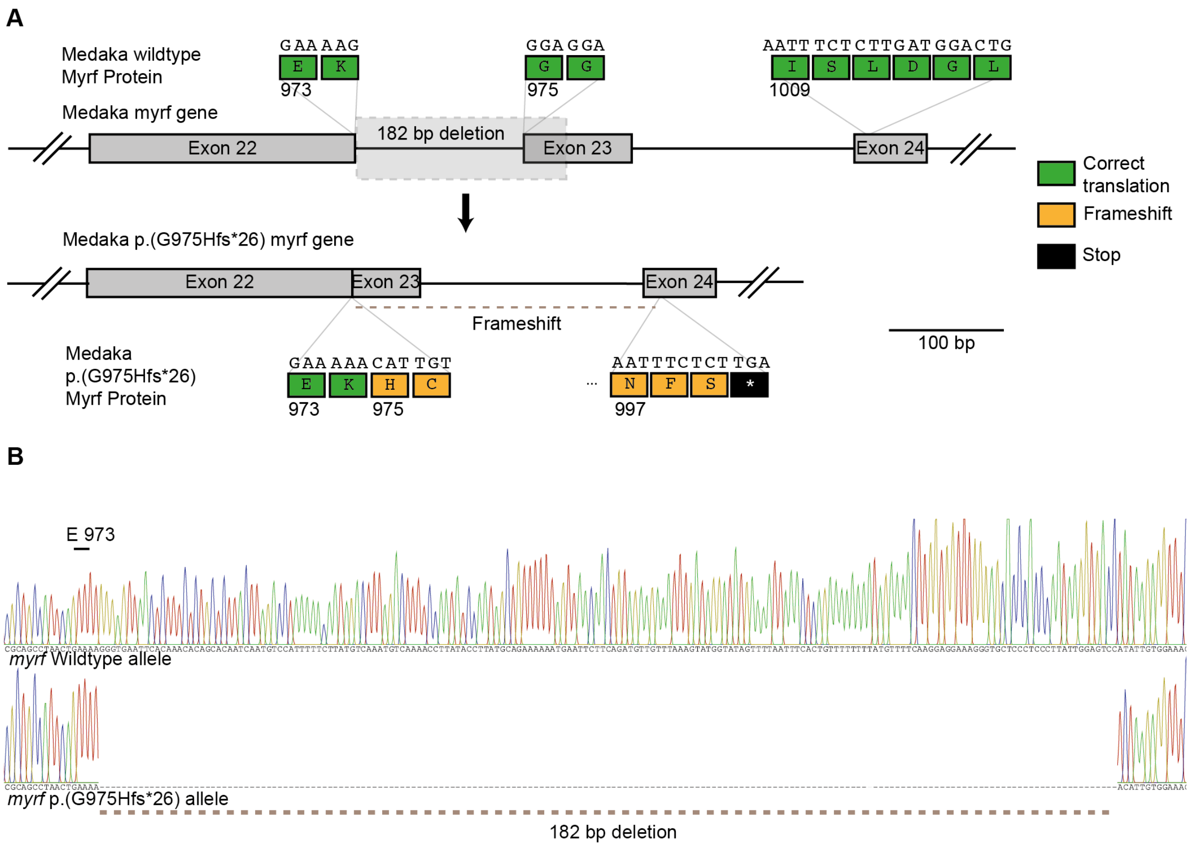

Fig. S4. Detailed scheme and sequencing of the medaka myrf p.(G975Hfs*26) allele. (**A**) Medaka *myrf* mutant allele recovered after CRISPR-Cas9 based targeting, including a frameshifting 182 bp deletion with a predicted protein-level p.(G975Hfs*26) allele resulting in a PTC. (**B**) Sanger sequencing confirmed the 182 bp deletion allele.

Disease Models & Mechanisms • Supplementary information

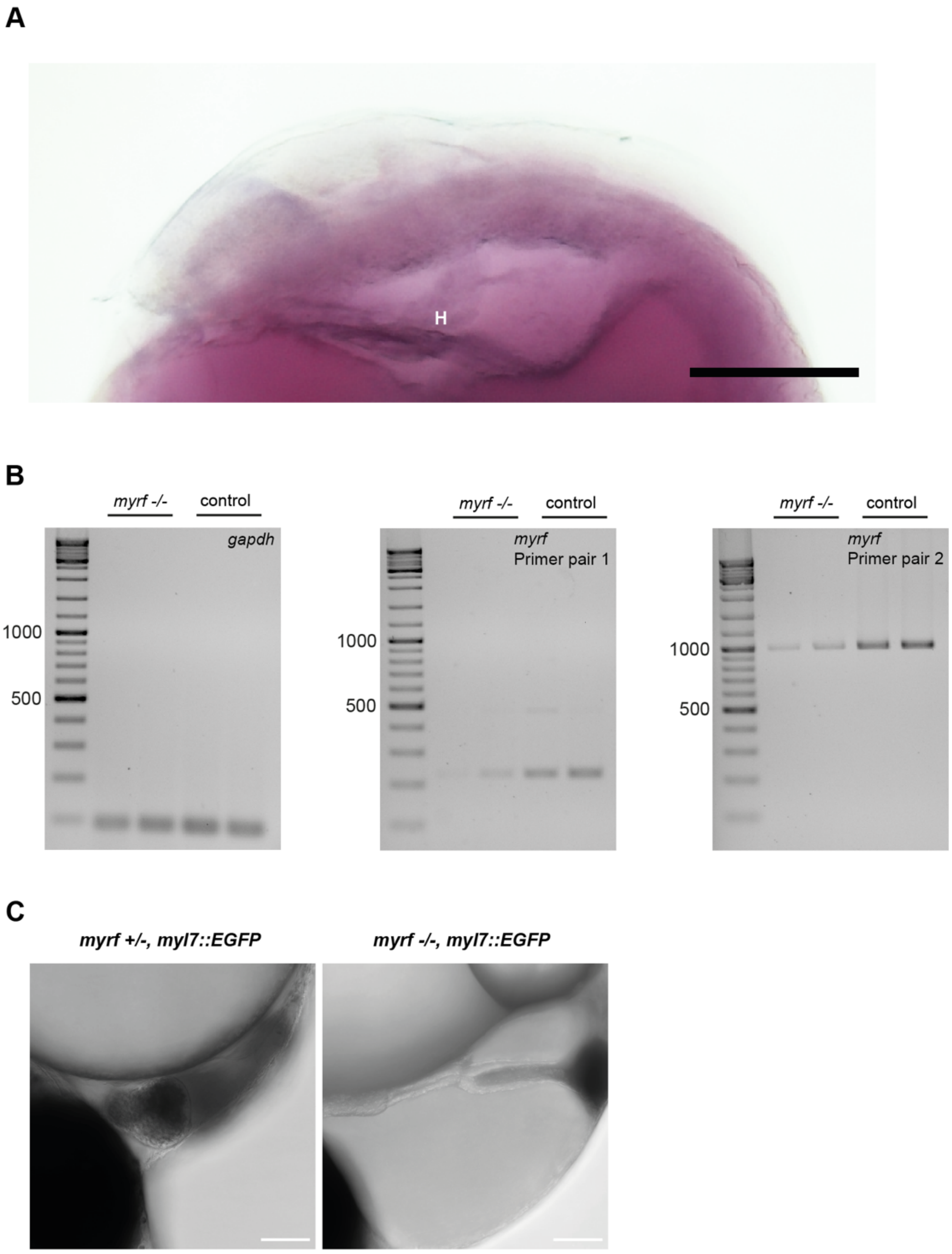

Fig. S5. Expression of *myrf* in wild-type and *myrf* -/- medaka embryos and bright-field images of the *myrf* mutant phenotype. (**A**) In situ hybridization for *myrf* mRNA in medaka embryonic stage 24, H: indicates the tubular heart, scale bar is 200 µm. (**B**) RT-PCR on *myrf* transcript in *myrf* mutant (two samples each from a pool of four embryos with characteristic *myrf*-related phenotypes) versus control (two samples each from a pool of four non-phenotypic siblings, positive control GAPDH). (**C**) Bright-field images corresponding to Fig. 3E, demonstrating an adhesion of myocardial muscle with the surrounding structures.

Disease Models & Mechanisms • Supplementary information

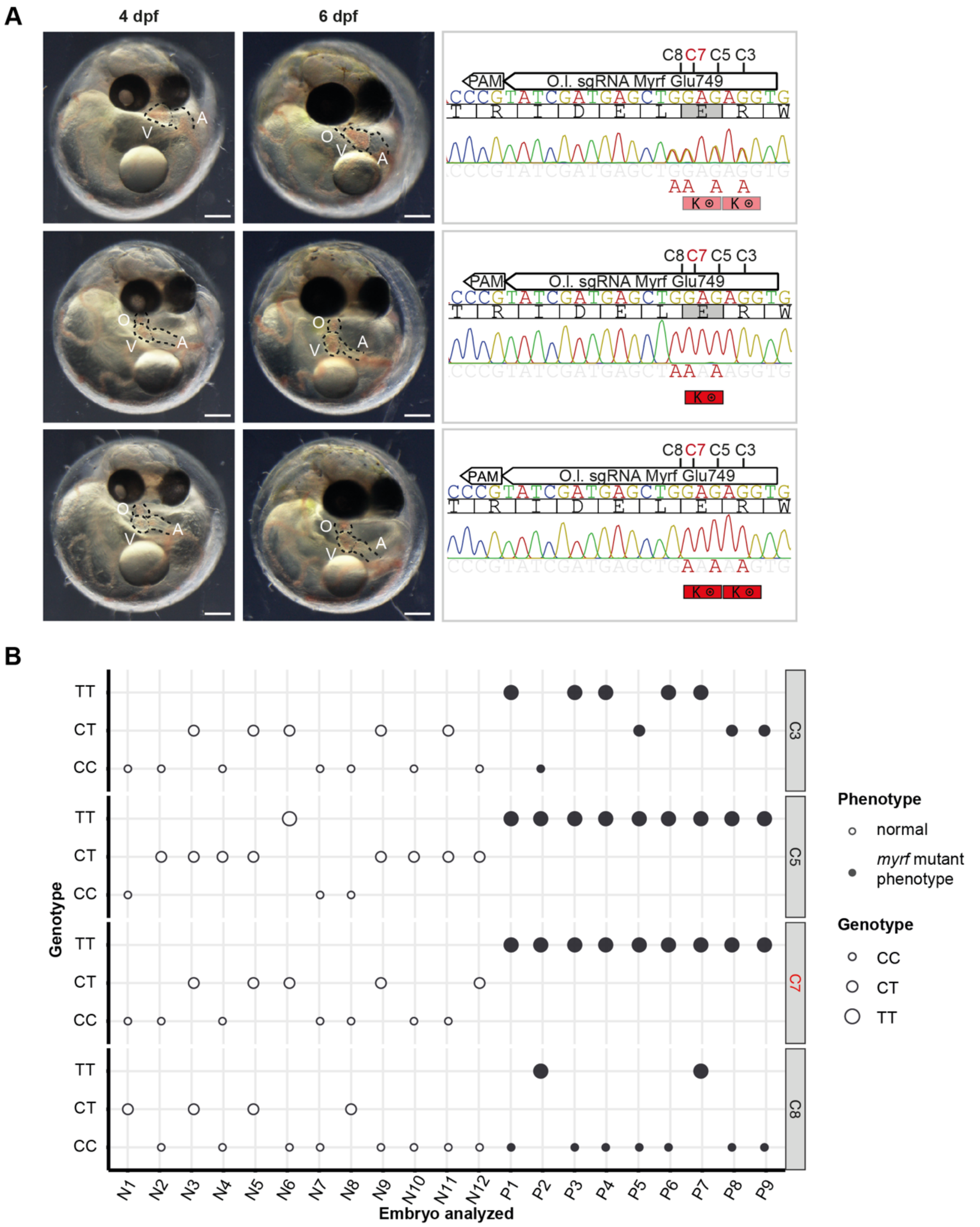

Fig. S6. Genotype-phenotype correlation in Myrf Glu749-edited F1 embryos. (**A**) Phenotypic embryos (bright-field images) at 4 dpf and 6 dpf obtained from Myrf Glu749 editant incross and genotypes (Sanger reads) at the sgRNA target site. Dashed lines indicate the heart circumference; A: atrium, V: ventricle, O: outflow tract. Bright-field images were equally adjusted for contrast in Fiji/ImageJ using the brightness and contrast function; scale bar: 200 µm. (**B**) Individual Sanger sequencing at the sgRNA Glu749 base editing window for the C-T edited nucleotides C3, C5, C7, and C8 (confer Fig. 4A).

Disease Models & Mechanisms • Supplementary information

Table S1. Two hundred genes selected from the literature subjected to *in silico* prioritization and filtering.

Gene	Study
Final gene selection based on individual gene ranking built on (1) number of clinical cases, (2) isolated cardiac defects reported in patients, and (3) biological, mechanistic plausibility based on in vitro evidence	
ADNP	(Ji et al., 2020; Sifrim et al., 2016)
AHNAK	(Jin et al., 2017)
ANKRD11	(Jin et al., 2017; Reuter et al., 2020; Sifrim et al., 2016)
CDC42BPA	(Reuter et al., 2020)
CLUH	(Jin et al., 2017)
COL11A1	(Liu et al., 2020; Xu et al., 2018)
DGUOK	(Jin et al., 2017)
DLX6	(Page et al., 2019)
DPH1	(Hawer et al., 2020)
DYRK1A	(Lee et al., 2020; Sifrim et al., 2016)
EVC2	(Liu et al., 2017; Priest et al., 2016)
GANAB	(Homsy et al., 2015; Jin et al., 2017)
IQGAP1	(Reuter et al., 2020)
KLHL26	(Samudrala et al., 2020)
LZTR1	(Jin et al., 2017)
MAPRE2	(Richter et al., 2020)
MED13L	(Ji et al., 2020)
MED25	(Haynes et al., 2020)
MINK1	(Jin et al., 2017)
MLF1	(Liu et al., 2020)
MYRF	(Jin et al., 2017)
NAB1	(Richter et al., 2020)
NADSYN1	(Szot et al., 2019)
NEXMIF	(Reuter et al., 2020)
PACS1	(Sifrim et al., 2016)
PCM1	(Jin et al., 2017; Page et al., 2019)

Disease Models & Mechanisms • Supplementary information

PYGL	(Jin et al., 2017)
RABGAP1L	(Homsy et al., 2015; Jin et al., 2017)
RIOK1	(Jin et al., 2017; Liu et al., 2020)
RPL29	(Richter et al., 2020)
SMG7	(Liu et al., 2020)
TMLHE	(Liu et al., 2020)
U2SURP	(Jin et al., 2017)
WDR26	(Richter et al., 2020)
WDR37	(Hay et al., 2020)
XPNPEP2	(Liu et al., 2020)
ZFP36L2	(Szot et al., 2018)
The following genes were excluded based on (1) low conservation or (2) the presence of two highly conserved orthologs	
ACTB	(Jin et al., 2017)
AK7	(Liu et al., 2020)
ANK3	(Jin et al., 2017)
CACNA1A	(Jin et al., 2017)
CDKL5	(Liu et al., 2020)
CEP57	(Liu et al., 2020)
COL6A2	(Xu et al., 2018)
CYP21A2	(Jin et al., 2017)
DPH2	(Hawer et al., 2020)
DSCAML1	(Jin et al., 2017)
EHMT1	(Priest et al., 2016; Xu et al., 2018)
EVC	(Priest et al., 2016)
FGD5	(Reuter et al., 2020)
ITSN2	(Jin et al., 2017)
KANSL1	(Dasouki et al., 2020; Jin et al., 2017)
KMT2C	(Jin et al., 2017; Szot et al., 2018)
MYO7B	(Jin et al., 2017)
NME8	(Liu et al., 2020)
NPHP1	(Dasouki et al., 2020)

NSD1	(Jin et al., 2017; Priest et al., 2016; Sifrim et al., 2016)
PLCB1	(Dasouki et al., 2020)
POGZ	(Jin et al., 2017; Reuter et al., 2020)
PPL	(Homsy et al., 2015)
PURA	(Reuter et al., 2020)
RBFOX2	(Homsy et al., 2015)
RNF38	(Liu et al., 2020)
RPL5	(Jin et al., 2017)
RYR3	(Jin et al., 2017)
VASP	(Reuter et al., 2020)
The following genes were excluded based on (1) previous knockout *in vivo* data reporting developmental cardiac phenotypes or (2) lack of an ortholog in medaka	
ABL1	(Bravo-Gil et al., 2019)
ABL1M1	(Fotiou et al., 2019)
ACAP12	(Jin et al., 2017)
ADAM17	(Priest et al., 2016)
ARL13B	(Liu et al., 2020)
ATE1	(Priest et al., 2016)
B9D1	(Liu et al., 2020)
BBS2	(Priest et al., 2016)
BCOR	(Richter et al., 2020)
BMPR1A	(Demal et al., 2019)
BRAF	(Jin et al., 2017)
C5ORF42	(Xu et al., 2018)
CAD	(Homsy et al., 2015)
CAMTA2	(Page et al., 2019)
CATSPERG	(Liu et al., 2020)
CCDC39	(Liu et al., 2017)
CCT2	(Liu et al., 2020)
CD160	(Priest et al., 2016)
CDK13	(Jin et al., 2017; Sifrim et al., 2016)

Disease Models & Mechanisms • Supplementary information

CFAP54	(Liu et al., 2020)
CFH	(Liu et al., 2020)
CHD4	(Sifrim et al., 2016)
CHD7	(Sifrim et al., 2016)
CHRD	(Priest et al., 2016)
CITED2	(Dianatpour et al., 2020)
CLASP1	(Liu et al., 2020)
CLUAP1	(Liu et al., 2020)
CNOT1	(Szot et al., 2018)
COL6A1	(Xu et al., 2018)
CREBBP	(Richter et al., 2020)
CTNNB1	(Jin et al., 2017)
CUL4B	(Jin et al., 2017)
DAND5	(Liu et al., 2017)
DIAPH2	(Liu et al., 2020)
DISC1	(Liu et al., 2020)
DNAAF2	(Liu et al., 2020)
DNAI1	(Liu et al., 2020)
DSG2	(Reuter et al., 2020)
DTNA	(Homsy et al., 2015; Jin et al., 2017)
DYNC2LI1	(Liu et al., 2020)
ELN	(Jin et al., 2017)
FBN1	(Homsy et al., 2015)
FLNA	(Liu et al., 2020)
FLT4	(Jin et al., 2017; Reuter et al., 2020; Szot et al., 2018)
FN1	(Wang et al., 2020b)
FNIP1	(Richter et al., 2020)
FOXH1	(Liu et al., 2020)
FOXP1	(Ji et al., 2020)
FRYL	(Jin et al., 2017)
GLI3	(Xu et al., 2018)

Disease Models & Mechanisms • Supplementary information

GPBAR1	(Jin et al., 2017)
GPR161	(Liu et al., 2020)
HAND2	(Cohen et al., 2020)
HIRA	(Richter et al., 2020)
HSPA2	(Liu et al., 2020)
HSPG2	(Xu et al., 2018)
IFT140	(Priest et al., 2016)
IFT43	(Liu et al., 2020)
JAG1	(Homsy et al., 2015)
JPH2	(Richter et al., 2020)
KDR	(Reuter et al., 2020)
KIAA0196	(Jin et al., 2017)
KLF13	(Li et al., 2020)
KLF2	(Richter et al., 2020)
KMT2A	(Jin et al., 2017; Sifrim et al., 2016)
KMT2D	(Homsy et al., 2015; Jin et al., 2017; Reuter et al., 2020)
KRT13	(Homsy et al., 2015)
LRP1	(Jin et al., 2017)
LRRC6	(Liu et al., 2020)
MAP2K1	(Wang et al., 2020a)
MEF2	(Richter et al., 2020)
MTHFR	(Liu et al., 2020)
MYH11	(Reuter et al., 2020)
MYH6	(Homsy et al., 2015)
NALCN	(Jin et al., 2017)
NGFR	(Jin et al., 2017)
NIPBL	(Reuter et al., 2020; Xu et al., 2018)
NOTCH1	(Homsy et al., 2015)
NR2F2	(Reuter et al., 2020; Xu et al., 2018)
NR3C1	(Dasouki et al., 2020)
NR6A1	(Jin et al., 2017)

Disease Models & Mechanisms • Supplementary information

NRK	(Liu et al., 2020)
NSD2	(Ji et al., 2020)
NUP188	(Muir et al., 2020)
OFD1	(Richter et al., 2020)
OR52M1	(Liu et al., 2020)
PBX1	(Szot et al., 2018)
PDZK1	(Priest et al., 2016)
PHKA1	(Liu et al., 2020)
POC1B	(Liu et al., 2020)
PRKAR1A	(Liu et al., 2020)
PRKD1	(Sifrim et al., 2016)
PRRC2B	(Jin et al., 2017)
PRX	(Liu et al., 2020)
PTEN	(Jin et al., 2017)
PTPN11	(Homsy et al., 2015; Reuter et al., 2020; Sifrim et al., 2016)
PTPRJ	(Priest et al., 2016)
PVRL2	(Liu et al., 2017)
RAF1	(Jin et al., 2017)
RIT1	(Jin et al., 2017)
RYR1	(Page et al., 2019)
SALL1	(Reuter et al., 2020)
SCN10A	(Jin et al., 2017)
SHANK3	(Xu et al., 2018)
SLC4A1	(Liu et al., 2020)
SLIT2	(Fotiou et al., 2019)
SLIT3	(Fotiou et al., 2019)
SMAD5	(Richter et al., 2020)
SMARCC1	(Reuter et al., 2020; Richter et al., 2020)
SMC1A	(Xu et al., 2018)
SNX8	(Mastromoro et al., 2020)
SRCAP	(Priest et al., 2016)

Disease Models & Mechanisms • Supplementary information

TAB2	(Sifrim et al., 2016)
TAF1	(Morton et al., 2020)
TBX1	(Page et al., 2019)
TEAD2	(Szot et al., 2018)
TEK	(Szot et al., 2018)
TFAP2B	(Szot et al., 2018)
TIE1	(Szot et al., 2018)
TLN2	(Reuter et al., 2020)
TMEM260	(Liu et al., 2020)
TPCN1	(Reuter et al., 2020)
TRPM4	(Reuter et al., 2020)
TSC1	(Jin et al., 2017)
UBXN10	(Reuter et al., 2020)
UPF2	(Szot et al., 2018)
VCAN	(Priest et al., 2016)
WHSC1	(Jin et al., 2017)
ZEB2	(Homsy et al., 2015)
ZFPM1	(Page et al., 2019)
ZFPM2	(Priest et al., 2016)
ZNF407	(Liu et al., 2020)
ZNF528	(Liu et al., 2017)
ZNF717	(Page et al., 2019)

Table S2. Human phenotypes associated with 12 candidate genes selected for CRISPR-Cas9-mediated validation and indication of gene function.

Candidate Gene	Human phenotype association	Gene function
SMG7	6 cases with variants in a non-syndromic TGA cohort (p.R411Q, p.P767L, p.P819A, p.R1136W) (Liu et al., 2020)	Nonsense mediated mRNA decay factor (Wittkopp et al., 2009)

Disease Models & Mechanisms • Supplementary information

DYRK1A	3 Protein truncating and one missense *de novo* variant in a syndromic CHD cohort (Sifrim et al., 2016)<br>1 case with a stop-gain *de novo* variant with CHD and EM (p.E396ter) (Lee et al., 2020)	Serin/Threonine kinase (Laham et al., 2021)
RABGAP1L	2 cases with *de novo* variants and LVO (p.S496G, 1 splice mutation), 1 case with AVC and a loss of function heterozygous splice mutation inherited from a parent (Jin et al., 2017)	Intracellular transport (Qu et al., 2016)
IQGAP1	1 case with an inherited stop gain variant TOF, PS, DORV + EM (p.(Arg766*)) (Reuter et al., 2020)<br>1 case with an inherited stop-gain variant and TOF, ASD, bicuspid pulmonic valve p.(Tyr103*) (Reuter et al., 2019)<br>2 cases (1 TOF, 1 TGA) with *de novo* loss of function variants (Petrovski et al., 2019)<br>1 case with a frameshift variant inherited from a parent and LVO + NDD (p.T1614fs), 2 cases with *de novo* missense variants and isolated CHD (p.R1130W, p.R1427L) (Jin et al., 2017)	Scaffolding protein (Abel et al., 2015)
CDC42BPA	1 patient with a *de novo* stop-gain variant and TOF (Gln24*) (Reuter et al., 2020)<br>1 patient with an inherited frameshift deletion variant and CTD (p.K493fs) (Jin et al., 2017)	Serin/threonin-protein kinase; downstream effector of CDC42; CDC42 functions in cell migration, cytoskeletal remodeling, cell cycle control and is implicated in cardiac OFT development (Fritz et al.,

Disease Models & Mechanisms • Supplementary information

		2019; Leung et al., 1998; Wilkinson et al., 2005)
MYRF	3 cases with a *de novo* missense variant and LVO + EM (p.Q394H, p.F378S, p.L470V) (Jin et al., 2017)<br>16 patients with CHD and human cardiac-urogenital syndrome (c.2336+1G>A, p.(Arg840*), p.(Thr419Argfs*14), p.(Gly81Trpfs*45), p.(Gly435Arg), p.(Val679Ala), p.(Arg695His), c.1904–1G>A, p.(Gln596*), p.(Phe387Ser), p.(Gln403His), p.(Leu479Val), p.(Arg1040Gly), p.(Glu1081Glyfs*5), p.(Gly117Valfs*31))<br>(Rossetti et al., 2019)	Myelin regulatory factor, transcription factor implicated in CNS myelination (Emery et al., 2009)
KLHL26	1 case with an inherited frameshift allele and CHD + EM (p.V80fs) (Jin et al., 2017)<br>Family with an inherited missense variant and 10/17 members affected with Ebsteins'anomaly and Left ventricular non compaction (p.R237C) (Samudrala et al., 2020)	Potential role in ubiquitin-mediated protein degradation<br>(Samudrala et al., 2020)
DLX6	7 variants in a cohort with non-syndromic TOF (Page et al., 2019)	Transcription factor (Charité et al., 2001)

Disease Models & Mechanisms • Supplementary information

MAPRE2	Enrichment of *de novo* mutations in enhancer regions of MAPRE2 in a CHD cohort (Richter et al., 2020)<br>2 cases with inherited stop-gain variants and isolated HTX (p.S28X) / CTD + EM + NDD (p.Q307X); 1 case with a *de novo* missense variant and CTD + EM (p.H291Q) (Jin et al., 2017)<br>Association with congenital symmetric circumferential skin creases type 2 (Feng et al., 2020), rare cases are reported with CHD (Basel-Vanagaite et al., 2012; Isrie et al., 2015)<br>Suggested as a susceptibility gene for Brugada syndrome (Barc et al., 2022)	Microtubule associated protein, microtubule organization, cell motility, adhesion (Brüning-Richardson et al., 2011; Fagerberg et al., 2014; Feng et al., 2020; Goldspink et al., 2013)
MLF1	1 missense, 1 stop-gain variant and isolated non-syndromic TGA (p.R138X, p.F93S) (Liu et al., 2020)	Myeloid leukemia factor 1, cilia-related (Liu et al., 2020)
ANKRD11	5 cases with protein truncating variants in a syndromic CHD cohort (Sifrim et al., 2016)<br>1 case with a *de novo* stop-gain variant AVSD + EM (Pro1747Argfs*49) (Reuter et al., 2020)<br>2 cases with *de novo* synonymous variants and CTD + NDD (p.S2553S) / CTD + EM (p.I2527I) (Jin et al., 2017)<br>Mutations cause KGB syndrome, which is associated with CHD, mainly AVSD (Digilio et al., 2022; Ockeloen et al., 2015; Swols et al., 2017)	Chromatin modifier, regulation of histone acetylation via interaction with histone deacetylase 3 (HDAC3) (Gallagher et al., 2015; Roth et al., 2021)

Disease Models & Mechanisms • Supplementary information

EVC2	Deletion in a patient with AVSD (Priest et al., 2016)<br>Mutations cause Ellis-van-Crefeld syndrome. 2/3 children have CHD, predominantly AVCD (Pugnaloni et al., 2020)<br>2 cases with inherited frameshift variants and CHD + EM (p.T462fs) / TGA (p.V968fs); 1 case with an inherited stop-gain variant (p.Q1012X) (Jin et al., 2017)	Cilia transmembrane protein, modulates Indian hedgehog signaling (Blair et al., 2011)

ASD: Atrial septal defect, AVCD: Atrioventricular canal defect, AVSD: Atrioventricular septal defect, CHD: Congenital heart disease, CTD: Conotruncal defect, DORV: Double outlet right ventricle, EM: Extracardiac manifestations, NDD: Neurodevelopmental disorder, PS: Pulmonary stenosis, TGA: Transposition of the great arteries, TOF: Teratology of Fallot

Disease Models & Mechanisms • Supplementary information

Table S3. sgRNas used in this work.

sgRNA	Target site [PAM] 5′-3′	Application	Editing events (Sanger)	Estimated efficiency (TIDE)
ankrd11 T1	ACTGAATGGCACAGCCTGAT[TGG]	Cas9	yes	84.8%
ankrd11 T2	CCAGTGGGCCTATTTTGATC[TGG]	Cas9	yes	84.6%
cdc42bpab T1	GACGGACCGGCCCAGTCTAA[CGG]	Cas9	yes	20.4%
cdc42bpab T2	CAGAGACCACCAGTCGCACT[CGG]	Cas9	yes	82.3%
dlx6 T1	AATGGGGAGATTCGTTTTAA[CGG]	Cas9	no	13.8%
dlx6 T2	CTGGATGGGGTCACTCTCGT[GGG]	Cas9	yes	50.5%
dyrk1a T1	GGCTAGGTCCATTATGTGAT[TGG]	Cas9	yes	56.3%
dyrk1a T2	GCTCCACTCCATCTTAGGTG[TGG]	Cas9	yes	60.9%
evc2 T1	CCTCGCTGGTCATTGCAACC[TGG]	Cas9	yes	52.1%
evc2 T2	TTGTAGCTATCTGAACGGCG[AGG]	Cas9	yes	34.6%
iqgap1 T1	CGTGGGCCATGGCCTGCTCT[GGG]	Cas9	yes	67.8%
iqgap1 T2	CATTCCTCACCAGGTACCCA[CGG]	Cas9	yes	85.8%
klhl26 T1	GAAGAAGATGAGTCGCTCCA[GGG]	Cas9	yes	81.0%
klhl26 T2	CCTTCAGCTCAATGGTATCT[TGG]	Cas9	yes	53.6%
mapre2 T1	CAGGCATGATATTATTGCCT[GGG]	Cas9	yes	81.4%
mapre2 T2	GAACACATTAAAGTCGGCCC[TGG]	Cas9	yes	89.3%
mlf1 T1	GTTATGACTTACTCAAAAGT[GGG]	Cas9	no	7.4%
mlf1 T2	CGACGACATGTCCTCTGTCT[TGG]	Cas9	yes	75.5%

Disease Models & Mechanisms • Supplementary information

sgRNA	Target site [PAM] 5′-3′	Application	Editing events (Sanger)	Estimated efficiency (TIDE)
myrf T1	CCTTATTGGAGTCCATATTG[TGG]	Cas9	yes	75.6%
myrf T2 (Phe437)	GGAGAAGTTGAAGCCTTTGT[CGG]	Base editing	yes	Cf. Fig. 4
myrf T3 (Gln453)	TTCCAGGTTACTGTGTACGT[CGG]	Base editing	yes	Cf. Fig. 4
myrf T4	GGAAACCCGTATCGATGAGC[TGG]	Cas9	yes	NA
myrf T5	AAACAGTGTGGAACCATCAA[AGG]	Cas9	yes	NA
myrf T6 (Glu749)	ACCTCTCCAGCTCATCGATA[CGG]	Base editing	yes	Cf. Fig. 4
oca2 T1 (Q333)	GAAACCCAGGTGGCCATTGC[AGG]	Base editing	yes	NA
oca2 T3	TTGCAGGAATCATTCTGTGT[GGG]	Cas9	yes	NA
rabgap1l T1	AGTGACAATGAAATCTCCAG[CGG]	Cas9	yes	69.8%
rabgap1l T2	GCTCCTGGAAGTGGGCCCAC[AGG]	Cas9	yes	91.4%
smg7 T1	TGCTCCAGCGCCGACGGGCC[CGG]	Cas9	yes	74.8%
smg7 T2	GAAGGCGCTGTCCAAGGCCC[TGG]	Cas9	yes	39.5%
tbx1 T1	AGAAGCATGTAGTCCGCCAT[GGG]	Cas9	yes	40.2%
tbx1 T2	GAAAGCGGATCCTGCCACAC[CGG]	Cas9	yes	69.3%

NA: not analyzed as the locus was not a target in the CRISPR-Cas9-screen (Fig. 1) or the *myrf* base editing experiments (Fig. 4). The following sgRNAs were taken from previous work: MYRF T1 (Hammouda et al., 2021), OCA2 T1 (Q333) and OCA2 T3 (Lischik et al., 2019).

Table S4. Sequencing primers.

Primer name	Primer sequence 5′-3′
smg7_T1_seq_fwd	CCTCCCCAGCTTCACCATTT
smg7_T1_seq_rev	GCTCCTGGACCAATCAGGAG
smg7_T2_seq_fwd	GGAAAAGCAAAGCTCCGTGA
smg7_T2_seq_rev	CAGGTCAGCCCTACAACCAG
dyrk1a_T1_seq_fwd	TCCAGAGTCGCTTCTACCGT
dyrk1a_T1_seq_rev	GCTGGAGGCTTATACTACAAAGA
dyrk1a_T2_seq_fwd	AGATGGCAAAAGGGTGAGTTGA
dyrk1a_T2_seq_rev	AGATTGTTTTGAATCGCCTGCA
rabgap1l_T1_seq_fwd	ACTTTACCTCTTTGTGCCAAAACT
rabgap1l_T1_seq_rev	CCACTTTTTATCTGGGCGGC
rabgap1l_T2_seq_fwd	ACATTTAGCTCTTATTTTGCAATGGG
rabgap1l_T2_seq_rev	AGTTCAAGCCATTGCAGCTG
iqgap1_T1_seq_fwd	TTTCCCCCGAAGAACTGGAC
iqgap1_T1_seq_rev	TGGAAGATCCCAACGTGTGA
iqgap1_T2_seq_fwd	CATGGGTGGAACCAGAGGAC
iqgap1_T2_seq_rev	CTTACGAGCTTGGTGCATGC
cdc42bpab_T1_seq_fwd_2	TCACCCCAATCCAGAAACTCC
cdc42bpab_T1_seq_rev_2	GGCAGCTTTTTACACTCCCG
cdc42bpab_T2_seq_fwd	AGTCCTCAATGCCGTTCTGG

Disease Models & Mechanisms • Supplementary information

Primer name	Primer sequence 5′-3′
cdc42bpab_T2_seq_rev	AGCAGAAGTCACAGAGCTGT
klhl26_T1_seq_fwd	ACATCAGAGCGAATGAGGGT
klhl26_T1_seq_rev	CGAGCTCTGCGAAGAATTCC
klhl26_T2_seq_fwd	TCCAATGTGCAGGCAGGTC
klhl26_T2_seq_rev	TCGTGATTCTGAAGACAATGTGG
dlx6_T1_seq_fwd	GCTCAGCTTGAAACGTTGCA
dlx6_T1_seq_rev	TCGTGCAATTGTGATTTCAATCGA
dlx6_T2_seq_fwd	GACTGACCCAAACGCAGGTA
dlx6_T2_seq_rev	AAGTCCTCTCGTTTGTGGGG
mapre2_T1_seq_fwd_2	GGGATGGCGGTCAACGTATA
mapre2_T1_seq_rev_2	GAGCTCTGCAGGTTCGTTCA
mapre2_T2_seq_fwd_2	TGCAGACTGTGTCCACTTCC
mapre2_T2_seq_rev_2	AACAAACCCGCTGTCCTGAA
mlf1_T1_seq_fwd	TGACTGATTTGCTGGGTAAATTGT
mlf1_T1_seq_rev	TCGTTTTCAAAAGATTTACAACACTG
mlf1_T2_seq_fwd	TCTGCCAGTAGGTGCTACCA
mlf1_T2_seq_rev	GCCATGTTCAGGACCAGGAA
mlf1_T2_seq_fwd_2	GATCAAGGAGACTCTGCGGG
mlf1_T2_seq_rev_2	GGTAAGCAGCATGGGGTTCA
ankrd11_T1_seq_fwd	TCAGGTTGCAGCACAGCATA

Disease Models & Mechanisms • Supplementary information

Primer name	Primer sequence 5′-3′
ankrd11_T1_seq_rev	AACTGACGTGCATTGAAGCG
ankrd11_T2_seq_fwd	CCACAGAGGACACACGGAAG
ankrd11_T2_seq_rev	GTAAGTTGGGGAGCAGGCAT
evc2_T1_seq_fwd	GAAATGTCCACACTGCTGCG
evc2_T1_seq_rev	CGTCAGATTTTTGTTTAATTGAATGCA
evc2_T2_seq_fwd	GGAGAGCCTGCAGGAGAGAA
evc2_T2_seq_rev	TATGACCTGAGCCAGCTCCT
tbx1_T1_seq_fwd	AGTGTGTTTGGAATGAGCCTCT
tbx1_T1_seq_rev	ATCAGTAGACACCGCAGCAC
tbx1_T2_seq_fwd	AAGCGCGTTTGAACTATTATTAGT
tbx1_T2_seq_rev	CGTTGTCATCCAGCAGGTTG
myrf_T1_seg_fwd	GGTCCACCAACCGCCTGCC
myrf_T1_seq_rev	AGCATCCTAGCATTGCAGCC
myrf_T2_T3_seq_F	TCCGATGCCAACGTACCG
myrf_T2_T3_seq_R	TTTATGGAGAACAATGCATTCTGAGT
myrf_T4_T6_seq_F	GAGTTATTGCCCAGGAGGTTCA
myrf_T4_T6_seq_R	CTTGCTCCCAGGCTTGACTG
myrf_T5_seq_F	CTTCACAAGTAGCGTTTGGGC
myrf_T5_seq_R	AGACAGAGATAAACGAAGGTGAT

Disease Models & Mechanisms • Supplementary information

References

Abel, A. M., Schuldt, K. M., Rajasekaran, K., Hwang, D., Riese, M. J., Rao, S., Thakar, M. S. and Malarkannan, S. (2015). IQGAP1: Insights into the function of a molecular puppeteer. *Mol Immunol* 65, 336–349.

Barc, J., Tadros, R., Glinge, C., Chiang, D. Y., Jouni, M., Simonet, F., Jurgens, S. J., Baudic, M., Nicastro, M., Potet, F., et al. (2022). Genome-wide association analyses identify new Brugada syndrome risk loci and highlight a new mechanism of sodium channel regulation in disease susceptibility. *Nat Genet* 54, 232–239.

Basel-Vanagaite, L., Sprecher, E., Gat, A., Merlob, P., Albin-Kaplanski, A., Konen, O., Solomon, B. D., Muenke, M., Grzeschik, K. and Sirota, L. (2012). New Syndrome of Congenital Circumferential Skin Folds Associated with Multiple Congenital Anomalies. *Pediatr Dermatol* 29, 89–95.

Blair, H. J., Tompson, S., Liu, Y.-N., Campbell, J., MacArthur, K., Ponting, C. P., Ruiz-Perez, V. L. and Goodship, J. A. (2011). Evc2 is a positive modulator of Hedgehog signalling that interacts with Evc at the cilia membrane and is also found in the nucleus. *Bmc Biol* 9, 14.

Bravo-Gil, N., Marcos, I., González-Meneses, A., Antiñolo, G. and Borrego, S. (2019). Expanding the clinical and mutational spectrum of germline ABL1 mutations-associated syndrome. *Medicine* 98, e14782.

Brüning-Richardson, A., Langford, K. J., Ruane, P., Lee, T., Askham, J. M. and Morrison, E. E. (2011). EB1 Is Required for Spindle Symmetry in Mammalian Mitosis. *Plos One* 6, e28884.

Charité, J., McFadden, D. G., Merlo, G., Levi, G., Clouthier, D. E., Yanagisawa, M., Richardson, J. A. and Olson, E. N. (2001). Role of Dlx6 in regulation of an endothelin-1-dependent, dHAND branchial arch enhancer. *Gene Dev* 15, 3039–3049.

Cohen, A. S. A., Simotas, C., Webb, B. D., Shi, H., Khan, W. A., Edelmann, L., Scott, S. A. and Singh, R. (2020). Haploinsufficiency of the basic helix–loop–helix transcription factor HAND2 causes congenital heart defects. *Am J Med Genet A* 182, 1263–1267.

Dasouki, M. J., Wakil, S. M., Al-Harazi, O., Alkorashy, M., Muiya, N. P., Andres, E., Hagos, S., Aldusery, H., Dzimiri, N. and Colak, D. (2020). New Insights into the Impact of Genome-Wide Copy Number Variations on Complex Congenital Heart Disease in Saudi Arabia. *Omics J Integr Biology* 24, 16–28.

Demal, T. J., Heise, M., Reiz, B., Dogra, D., Brænne, I., Reichenspurner, H., Männer, J., Aherrahrou, Z., Schunkert, H., Erdmann, J., et al. (2019). A familial congenital heart disease with a possible multigenic origin involving a mutation in BMPR1A. *Sci Rep-uk* 9, 2959.

Dianatpour, S., Khatami, M., Heidari, M. M. and Hadadzadeh, M. (2020). Novel Point Mutations of CITED2 Gene Are Associated with Non-familial Congenital Heart Disease (CHD) in Sporadic Pediatric Patients. *Appl Biochem Biotech* 190, 896–906.

Digilio, M. C., Calcagni, G., Gnazzo, M., Versacci, P., Dentici, M. L., Capolino, R., Sinibaldi, L., Baban, A., Putotto, C., Alfieri, P., et al. (2022). Congenital heart defects in molecularly confirmed KBG syndrome patients. *Am J Med Genet A* 188, 1149–1159.

Emery, B., Agalliu, D., Cahoy, J. D., Watkins, T. A., Dugas, J. C., Mulinyawe, S. B., Ibrahim, A., Ligon, K. L., Rowitch, D. H. and Barres, B. A. (2009). Myelin gene regulatory factor is a critical transcriptional regulator required for CNS myelination. *Cell* 138, 172–185.

Fagerberg, L., Hallström, B. M., Oksvold, P., Kampf, C., Djureinovic, D., Odeberg, J., Habuka, M., Tahmasebpoor, S., Danielsson, A., Edlund, K., et al. (2014). Analysis of the Human Tissue-specific Expression by Genome-wide Integration of Transcriptomics and Antibody-based Proteomics*. *Mol Cell Proteomics* 13, 397–406.

Feng, J., Lan, X., Shen, J., Song, X., Tang, X., Xu, W., Ren, X., Zhang, H., Yu, G. and Wu, S. (2020). A de novo MAPRE2 variant in a patient with congenital symmetric circumferential skin creases type 2. *Mol Genetics Genom Medicine* 8, e1096.

Fotiou, E., Williams, S., Martin-Geary, A., Robertson, D. L., Tenin, G., Hentges, K. E. and Keavney, B. (2019). Integration of Large-Scale Genomic Data Sources With Evolutionary History Reveals Novel Genetic Loci for Congenital Heart Disease. *Circulation Genom Precis Medicine* 12, 442–451.

Fritz, K. R., Zhang, Y. and Ruest, L. B. (2019). Cdc42 activation by endothelin regulates neural crest cell migration in the cardiac outflow tract. *Dev Dynam* 248, 795–812.

Gallagher, D., Voronova, A., Zander, M. A., Cancino, G. I., Bramall, A., Krause, M. P., Abad, C., Tekin, M., Neilsen, P. M., Callen, D. F., et al. (2015). Ankrd11 Is a Chromatin Regulator Involved in Autism that Is Essential for Neural Development. *Dev Cell* 32, 31–42.

Goldspink, D. A., Gadsby, J. R., Bellett, G., Keynton, J., Tyrrell, B. J., Lund, E. K., Powell, P. P., Thomas, P. and Mogensen, M. M. (2013). The microtubule end-binding protein EB2 is a central regulator of microtubule reorganisation in apico-basal epithelial differentiation. *J Cell Sci* 126, 4000–4014.

Hawer, H., Mendelsohn, B. A., Mayer, K., Kung, A., Malhotra, A., Tuupanen, S., Schleit, J., Brinkmann, U. and Schaffrath, R. (2020). Diphthamide-deficiency syndrome: a novel human developmental disorder and ribosomopathy. *Eur J Hum Genet* 28, 1497–1508.

Hay, E., Henderson, R. H., Mansour, S., Deshpande, C., Jones, R., Nutan, S., Mankad, K., Young, R. M., Moosajee, M., Consortium, G. E. R., et al. (2020). Expanding the phenotypic spectrum consequent upon de novo WDR37 missense variants. *Clin Genet* 98, 191–197.

Haynes, D., Pollack, L., Prasad, C., Goobie, S., Colaiacovo, S., Wolfinger, T. and Lacassie, Y. (2020). Further delineation of Basel-Vanagaite-Smirin-Yosef syndrome: Report of three patients. *Am J Med Genet A* 182, 1785–1790.

Homsy, J., Zaidi, S., Shen, Y., Ware, J. S., Samocha, K. E., Karczewski, K. J., DePalma, S. R., McKean, D., Wakimoto, H., Gorham, J., et al. (2015). De novo mutations in congenital heart disease with neurodevelopmental and other congenital anomalies. *Science* 350, 1262–1266.

Isrie, M., Breuss, M., Tian, G., Hansen, A. H., Cristofoli, F., Morandell, J., Kupchinsky, Z. A., Sifrim, A., Rodriguez-Rodriguez, C. M., Dapena, E. P., et al. (2015). Mutations in Either TUBB or MAPRE2 Cause Circumferential Skin Creases Kunze Type. *Am J Hum Genetics* 97, 790–800.

Ji, W., Ferdman, D., Copel, J., Scheinost, D., Shabanova, V., Brueckner, M., Khokha, M. K. and Ment, L. R. (2020). De novo damaging variants associated with congenital heart diseases contribute to the connectome. *Sci Rep-uk* 10, 7046.

Jin, S. C., Homsy, J., Zaidi, S., Lu, Q., Morton, S., DePalma, S. R., Zeng, X., Qi, H., Chang, W., Sierant, M. C., et al. (2017). Contribution of rare inherited and de novo variants in 2,871 congenital heart disease probands. *Nat. Genet.* 49, 1593–1601.

Laham, A. J., Saber-Ayad, M. and El-Awady, R. (2021). DYRK1A: a down syndrome-related dual protein kinase with a versatile role in tumorigenesis. *Cell Mol Life Sci* 78, 603–619.

Lee, K.-S., Choi, M., Kwon, D.-W., Kim, D., Choi, J.-M., Kim, A.-K., Ham, Y., Han, S.-B., Cho, S. and Cheon, C. K. (2020). A novel de novo heterozygous DYRK1A mutation causes complete loss of DYRK1A function and developmental delay. *Sci Rep-uk* 10, 9849.

Leung, T., Chen, X. Q., Tan, I., Manser, E. and Lim, L. (1998). Myotonic dystrophy kinase-related Cdc42-binding kinase acts as a Cdc42 effector in promoting cytoskeletal reorganization. *Mol Cell Biol* 18, 130–40.

Li, W., Li, B., Li, T., Zhang, E., Wang, Q., Chen, S. and Sun, K. (2020). Identification and analysis of KLF13 variants in patients with congenital heart disease. *Bmc Med Genet* 21, 78.

Liu, L., Bu, H., Yang, Y., Tan, Z., Zhang, F., Hu, S. and Zhao, T. (2017). A Targeted, Next-Generation Genetic Sequencing Study on Tetralogy of Fallot, Combined With Cleft Lip and Palate. *J Craniofac Surg* 28, e351–e355.

Liu, X., Chen, W., Li, W., Priest, J. R., Fu, Y., Pang, K., Ma, B., Han, B., Liu, X., Hu, S., et al. (2020). Exome-Based Case-Control Analysis Highlights the Pathogenic Role of Ciliary Genes in Transposition of the Great Arteries. *Circ. Res.* 126, 811–821.

Mastromoro, G., Capalbo, A., Guido, C. A., Torres, B., Fabbretti, M., Traversa, A., Giancotti, A., Ventriglia, F., Bernardini, L., Spalice, A., et al. (2020). Small 7p22.3 microdeletion: Case report of Snx8 haploinsufficiency and neurological findings. *Eur J Med Genet* 63, 103772.

Morton, S. U., Agarwal, R., Madden, J. A., Genetti, C. A., Brownstein, C. A., López-Giráldez, F., Choi, J., Seidman, C. E., Seidman, J. G., Lyon, G. J., et al. (2020). Congenital Heart Defects Due to TAF1 Missense Variants. *Circulation Genom Precis Medicine* 13, e002843.

Muir, A. M., Cohen, J. L., Sheppard, S. E., Guttipatti, P., Lo, T. Y., Weed, N., Doherty, D., DeMarzo, D., Fagerberg, C. R., Kjærsgaard, L., et al. (2020). Bi-allelic Loss-of-Function Variants in NUP188 Cause a Recognizable Syndrome Characterized by Neurologic, Ocular, and Cardiac Abnormalities. *Am J Hum Genetics* 106, 623–631.

Ockeloen, C. W., Willemsen, M. H., Munnik, S. de, Bon, B. W. van, Leeuw, N. de, Verrips, A., Kant, S. G., Jones, E. A., Brunner, H. G., Loon, R. L. van, et al. (2015). Further delineation of the KBG syndrome phenotype caused by ANKRD11 aberrations. *Eur J Hum Genet* 23, 1176–1185.

Page, D. J., Miossec, M. J., Williams, S. G., Monaghan, R. M., Fotiou, E., Cordell, H. J., Sutcliffe, L., Topf, A., Bourgey, M., Bourque, G., et al. (2019). Whole Exome Sequencing Reveals the Major Genetic Contributors to Nonsyndromic Tetralogy of Fallot. *Circ. Res.* 124, 553–563.

Petrovski, S., Aggarwal, V., Giordano, J. L., Stosic, M., Wou, K., Bier, L., Spiegel, E., Brennan, K., Stong, N., Jobanputra, V., et al. (2019). Whole-exome sequencing in the evaluation of fetal structural anomalies: a prospective cohort study. *Lancet* 393, 758–767.

Priest, J. R., Osoegawa, K., Mohammed, N., Nanda, V., Kundu, R., Schultz, K., Lammer, E. J., Girirajan, S., Scheetz, T., Waggott, D., et al. (2016). De Novo and Rare Variants at Multiple Loci Support the Oligogenic Origins of Atrioventricular Septal Heart Defects. *PLoS Genet.* 12, e1005963.

Pugnaloni, F., Digilio, M. C., Putotto, C., Luca, E. D., Marino, B. and Versacci, P. (2020). Genetics of atrioventricular canal defects. *Ital J Pediatr* 46, 61.

Qu, F., Lorenzo, D. N., King, S. J., Brooks, R., Bear, J. E. and Bennett, V. (2016). Ankyrin-B is a PI3P effector that promotes polarized α5β1-integrin recycling via recruiting RabGAP1L to early endosomes. *Elife* 5, e20417.

Reuter, M. S., Jobling, R., Chaturvedi, R. R., Manshaei, R., Costain, G., Heung, T., Curtis, M., Hosseini, S. M., Liston, E., Lowther, C., et al. (2019). Haploinsufficiency of vascular endothelial growth factor related signaling genes is associated with tetralogy of Fallot. *Genet Med* 21, 1001–1007.

Reuter, M. S., Chaturvedi, R. R., Liston, E., Manshaei, R., Aul, R. B., Bowdin, S., Cohn, I., Curtis, M., Dhir, P., Hayeems, R. Z., et al. (2020). The Cardiac Genome Clinic: implementing genome sequencing in pediatric heart disease. *Genet. Med.* 22, 1015–1024.

Richter, F., Morton, S. U., Kim, S. W., Kitaygorodsky, A., Wasson, L. K., Chen, K. M., Zhou, J., Qi, H., Patel, N., DePalma, S. R., et al. (2020). Genomic analyses implicate noncoding de novo variants in congenital heart disease. *Nat Genet* 52, 769–777.

Rossetti, L. Z., Glinton, K., Yuan, B., Liu, P., Pillai, N., Mizerik, E., Magoulas, P., Rosenfeld, J. A., Karaviti, L., Sutton, V. R., et al. (2019). Review of the phenotypic spectrum associated with haploinsufficiency of MYRF. *Am. J. Med. Genet. A* 179, 1376–1382.

Roth, D. M., Baddam, P., Lin, H., Vidal-García, M., Aponte, J. D., Souza, S.-T. D., Godziuk, D., Watson, A. E. S., Footz, T., Schachter, N. F., et al. (2021). The Chromatin Regulator Ankrd11 Controls Palate and Cranial Bone Development. *Frontiers Cell Dev Biology* 9, 645386.

Samudrala, S. S. K., North, L. M., Stamm, K. D., Earing, M. G., Frommelt, M. A., Willes, R., Tripathi, S., Dsouza, N. R., Zimmermann, M. T., Mahnke, D. K., et al. (2020). Novel KLHL26 variant associated with a familial case of Ebstein's anomaly and left ventricular noncompaction. *Mol Genetics Genom Medicine* 8, e1152.

Sifrim, A., Hitz, M.-P., Wilsdon, A., Breckpot, J., Turki, S. H. A., Thienpont, B., McRae, J., Fitzgerald, T. W., Singh, T., Swaminathan, G. J., et al. (2016). Distinct genetic architectures for syndromic and nonsyndromic congenital heart defects identified by exome sequencing. *Nat. Genet.* 48, 1060–1065.

Swols, D. M., Foster, J. and Tekin, M. (2017). KBG syndrome. *Orphanet J Rare Dis* 12, 183.

Szot, J. O., Cuny, H., Blue, G. M., Humphreys, D. T., Ip, E., Harrison, K., Sholler, G. F., Giannoulatou, E., Leo, P., Duncan, E. L., et al. (2018). A Screening Approach to Identify Clinically Actionable Variants Causing Congenital Heart Disease in Exome Data. *Circ Genom Precis Med* 11, e001978.

Szot, J. O., Campagnolo, C., Cao, Y., Iyer, K. R., Cuny, H., Drysdale, T., Flores-Daboub, J. A., Bi, W., Westerfield, L., Liu, P., et al. (2019). Bi-Allelic Mutations in NADSYN1 Cause Multiple Organ Defects and Expand the Genotypic Spectrum of Congenital NAD Deficiency Disorders. *Am J Hum Genetics* 106, 129–136.

Wang, Q., Chen, P., Peng, Q., Liu, J., Huang, Y., Tang, Z., Liu, Y. and Yuan, H. (2020a). Identification of a de novo MAP2K1 gene variant in an affected patient with Cardio-facio-cutaneous syndrome. *Chin J Medical Genetics* 37, 567–569.

Wang, Y., Jiang, T., Tang, P., Wu, Y., Jiang, Z., Dai, J., Gu, Y., Xu, J., Da, M., Ma, H., et al. (2020b). Family-based whole-genome sequencing identifies compound heterozygous protein-coding and noncoding mutations in tetralogy of Fallot. *Gene* 741, 144555.

Wilkinson, S., Paterson, H. F. and Marshall, C. J. (2005). Cdc42–MRCK and Rho–ROCK signalling cooperate in myosin phosphorylation and cell invasion. *Nat Cell Biol* 7, 255–261.

Wittkopp, N., Huntzinger, E., Weiler, C., Saulière, J., Schmidt, S., Sonawane, M. and Izaurralde, E. (2009). Nonsense-mediated mRNA decay effectors are essential for zebrafish embryonic development and survival. *Mol. Cell. Biol.* 29, 3517–3528.

Xu, J., Wu, Q., Wang, L., Han, J., Pei, Y., Zhi, W., Liu, Y., Yin, C. and Jiang, Y. (2018). Next-generation sequencing identified genetic variations in families with fetal non-syndromic atrioventricular septal defects. *Int J Clin Exp Patho* 11, 3732–3743.
